# Trojan horselike T6SS effector TepC mediates both interference competition and exploitative competition

**DOI:** 10.1093/ismejo/wrad028

**Published:** 2024-01-10

**Authors:** Li Song, Lei Xu, Tong Wu, Zhenkun Shi, Hafiz Abdul Kareem, Zhuo Wang, Qingyun Dai, Chenghao Guo, Junfeng Pan, Mingming Yang, Xiaomeng Wei, Yao Wang, Gehong Wei, Xihui Shen

**Affiliations:** State Key Laboratory for Crop Stress Resistance and High-Efficiency Production, College of Natural Resources and Environment, Northwest A&F University, Yangling, Shaanxi 712100, China; Shaanxi Key Laboratory of Agricultural and Environmental Microbiology, College of Life Sciences, Northwest A&F University, Yangling, Shaanxi 712100, China; Shaanxi Key Laboratory of Agricultural and Environmental Microbiology, College of Life Sciences, Northwest A&F University, Yangling, Shaanxi 712100, China; Shaanxi Key Laboratory of Agricultural and Environmental Microbiology, College of Life Sciences, Northwest A&F University, Yangling, Shaanxi 712100, China; Shaanxi Key Laboratory of Agricultural and Environmental Microbiology, College of Life Sciences, Northwest A&F University, Yangling, Shaanxi 712100, China; Shaanxi Key Laboratory of Agricultural and Environmental Microbiology, College of Life Sciences, Northwest A&F University, Yangling, Shaanxi 712100, China; Shaanxi Key Laboratory of Agricultural and Environmental Microbiology, College of Life Sciences, Northwest A&F University, Yangling, Shaanxi 712100, China; Shaanxi Key Laboratory of Agricultural and Environmental Microbiology, College of Life Sciences, Northwest A&F University, Yangling, Shaanxi 712100, China; Shaanxi Key Laboratory of Agricultural and Environmental Microbiology, College of Life Sciences, Northwest A&F University, Yangling, Shaanxi 712100, China; Shaanxi Key Laboratory of Agricultural and Environmental Microbiology, College of Life Sciences, Northwest A&F University, Yangling, Shaanxi 712100, China; College of Plant Protection, Northwest A&F University, Yangling, Shaanxi 712100, China; State Key Laboratory for Crop Stress Resistance and High-Efficiency Production, College of Natural Resources and Environment, Northwest A&F University, Yangling, Shaanxi 712100, China; Shaanxi Key Laboratory of Agricultural and Environmental Microbiology, College of Life Sciences, Northwest A&F University, Yangling, Shaanxi 712100, China; State Key Laboratory for Crop Stress Resistance and High-Efficiency Production, College of Natural Resources and Environment, Northwest A&F University, Yangling, Shaanxi 712100, China; Shaanxi Key Laboratory of Agricultural and Environmental Microbiology, College of Life Sciences, Northwest A&F University, Yangling, Shaanxi 712100, China; Shaanxi Key Laboratory of Agricultural and Environmental Microbiology, College of Life Sciences, Northwest A&F University, Yangling, Shaanxi 712100, China

**Keywords:** Type VI secretion system (T6SS), Siderophore, DNase, bacterial competition, cheater

## Abstract

The type VI secretion system (T6SS) is a bacterial weapon capable of delivering antibacterial effectors to kill competing cells for interference competition, as well as secreting metal ion scavenging effectors to acquire essential micronutrients for exploitation competition. However, no T6SS effectors that can mediate both interference competition and exploitation competition have been reported. In this study, we identified a unique T6SS-1 effector in *Yersinia pseudotuberculosis* named TepC, which plays versatile roles in microbial communities. First, secreted TepC acts as a proteinaceous siderophore that binds to iron and mediates exploitative competition. Additionally, we discovered that TepC has DNase activity, which gives it both contact-dependent and contact-independent interference competition abilities. In conditions where iron is limited, the iron-loaded TepC is taken up by target cells expressing the outer membrane receptor TdsR. For kin cells encoding the cognate immunity protein TipC, TepC facilitates iron acquisition, and its toxic effects are neutralized. On the other hand, nonkin cells lacking TipC are enticed to uptake TepC and are killed by its DNase activity. Therefore, we have uncovered a T6SS effector, TepC, that functions like a “Trojan horse” by binding to iron ions to provide a valuable resource to kin cells, whereas punishing cheaters that do not produce public goods. This lure-to-kill mechanism, mediated by a bifunctional T6SS effector, may offer new insights into the molecular mechanisms that maintain stability in microbial communities.

## Introduction

Microbial interactions range from cooperative behaviors to intense competition [1-4]. Although cooperative behaviors have been observed at nearly every taxonomic level, cooperators in microbial communities are susceptible to exploitation by cheaters [5-11]. Cheating occurs when an individual or group within a community exploits public goods without contributing to their production, creating a situation wherein the costs of producing public goods outweigh the benefits, leading to the collapse of the cooperative system over time [5-7, 11]. The most widespread type of cooperation is the secretion of so-called “public goods,” which incur high production costs but confer benefits on surrounding cells [5]. Such public goods include siderophores, extracellular enzymes, and biofilm matrices [1, 12, 13]. Siderophores are low-molecular-weight compounds that are secreted from cells and can bind to insoluble iron [14]. The potential outcomes are illustrated by the well-known scenario of the prisoner’s dilemma: cheaters with compatible receptors can exploit the siderophores generated by producers without contributing to production costs [5, 15]. The emergence of cheaters leads to the so-called “tragedy of the commons,” wherein public goods become exhausted and the cooperative behavior collapses [14-17]. Nevertheless, cooperation is ubiquitous, and several mechanisms have evolved to prevent cheaters from flourishing, such as kin discrimination, joint production of public and private goods, facultative cooperation regulated by quorum sensing, and partial privatization of public goods [10, 11, 15, 18-24]. Recently, several studies suggested that bacteria have evolved a toxin-based policing mechanism to prevent cheater infiltration and thereby enforce cooperation [8, 10, 11, 25, 26]. Although the idea that organisms as simple as bacteria exhibit punitive behavior is intriguing, the molecular mechanisms underlying policing remain largely unknown and even controversial [10, 26].

Most natural environments contain a diverse collection of microbial species, and there is a constant battle for survival as these species compete for limited resources [27]. The type VI secretion systems (T6SSs), which are contractile nano-machines that resemble phage tails, have been recognized as key players in intermicrobial interactions [28-32]. T6SSs were primarily considered to mediate interference competition by injection of toxic effectors that target essential components of bacterial cells, such as cell walls, membranes, and nucleic acids [33-36]. Recently, T6SSs were also reported to mediate exploitative competition by secretion of a panel of proteinaceous metallophores to enhance the ability of bacteria cells to acquire essential micronutrients such as manganese and zinc [37-41]. Generally, the delivery of effectors into target cells relies on contact-dependent piercing target cell membranes [32-34, 42-44]. However, T6SSs also exhibit contact-independent capabilities to promote metal acquisition and interbacterial competition [38, 39, 45-47]. Toxic or metal ion–binding T6SS effectors are secreted into the extracellular environment and taken up by bacterial cells via receptor-dependent mechanisms. However, T6SS-secreted proteinaceous metallophores may be exploited by both producer and nonproducer (cheater) strains as public goods in microbial communities. This raises the question: how do bacteria prevent cheaters from exploiting these public goods without contributing to their production?

*Yersinia pseudotuberculosis* (*Yptb*) is a Gram-negative enteric pathogen that causes a variety of gastrointestinal diseases in both humans and animals [48-50]. Four distinct T6SS clusters have been identified in the *Yptb* genome, and T6SS-3 has been reported to mediate interference competition through the secretion of toxic effectors Tce1 and CccR [46, 47], whereas T6SS-4 has been reported to mediate exploitative competition through the secretion of metal ion–binding proteins such as YezP and TssS [37, 51]. In this study, we uncovered a unique cheater policing mechanism employed by T6SS-1, which involves the secretion of a proteinaceous siderophore TepC (T6SS effector for policing Cheaters) that is armed with toxic DNase activity. This bifunctional T6SS-1 effector can mediate both exploitative and interference competition and employs a lure-to-kill mechanism to punish cheaters that exploit the TepC protein as public goods.

## Materials and methods

### Bacterial strains and growth conditions

Bacterial strains and plasmids used in this study are listed in Table S1. *Yptb* YPIII derivatives were grown in *Yersinia* lysogeny broth (YLB) (1% tryptone, 0.5% yeast extract, 0.5% NaCl) or M9 medium (Na_2_HPO_4_, 6 g·l^−1^; KH_2_PO_4_, 3 g·l^−1^; NaCl, 0.5 g·l^−1^; NH_4_Cl, 1 g·l^−1^; MgSO_4_, 1 mM; CaCl_2_, 0.1 mM; glucose 0.2%) at 30°C. *Escherichia coli* DH5α, S17-1, TG1, BL21(DE3), *Salmonella Typhimurium* SL1344, *Staphylococcus aureus* Mu50 or RN4220, *Acinetobacter baumannii*, *Klebsiella pneumoniae*, *Enterococcus xiangfangensis*, *Acinetobacter calcoaceticus*, and *Pseudomonas aeruginosa* PAO1 were cultured in LB broth at 37°C. Appropriate antibiotics were included in growth mediums, and their corresponding concentrations are Nalidixic acid (20 μg ml^−1^), Ampicillin (100 μg ml^−1^), Kanamycin (50 μg ml^−1^), Streptomycin (200 μg ml^−1^), Gentamicin (20 μg ml^−1^), Tetracycline (5 μg ml^−1^ for *Yptb* and 15 μg ml^−1^ for *E. coli*). Different mutant strains were grown in mediums with appropriate antibiotics, and the primers to generate mutant strains are listed in Table S2.

### Determination of intracellular ion content

To determine intracellular ion contents, 20 ml postexponential phase *Yptb* cultures were collected and washed with M9 twice. The collected pellets were lysed using BugBuster (Novagen, Madison, WI) with rotating incubation overnight at 4°C. The total protein for each sample was measured with NanoDrop ND-1000 spectrophotometer (Thermo Fisher Scientific). The samples were subjected to a 100-fold dilution in 2% molecular grade nitric acid, resulting in a final volume of 10 ml. The samples were analysed by inductively coupled plasma mass spectrometry (ICP-MS) (Varian 802-MS), and the results were corrected using suitable reference buffers and dilution factors. Triplicate cultures of each strain were analysed during a single experiment, and the experiment was repeated at least three times [46].

### DNase assay

Purified TepC protein was incubated with substrates (λ DNA or pUC19) in the reaction buffer containing 20 mM MES, 100 mM NaCl, 2 mM MgCl_2_, pH 8.0. The system was incubated at 37°C for 30 min, and results were detected by 0.7% agarose gel electrophoresis [46].

### Intraspecies and interspecies competition *in vitro*

Competition assays were conducted as described previously with minor modifications [38, 46]. The high initial starting density of the bacteria was used to allow bacteria to enter into a competitive state early on. For intraspecies competition assays, overnight-grown *Yptb* strains were washed and adjusted to OD_600_ of 1.0 and mixed with the initial donor-to-recipient ratio of 1:1. Similarly, for interspecies competition assays, overnight-grown *Yptb* donor strains and *E. coli* DH5α, *S. Typhimurium* SL1344, *Acinetobacter acetobacter*, or *E. xiangfangensis* recipient strains were washed with M9 medium and adjusted to OD_600_ of 1.0, and the donor and recipient strains were mixed 1:1. The mixtures were either spotted onto a 0.22-μm nitrocellulose membrane (Nalgene) on M9 agar plates at 30°C (for contact-dependent competition), or inoculated into 3 ml liquid medium at 30°C with shaking (for contact-independent competition). After competition, the samples were serially diluted and counted on YLB or LB plates containing appropriate antibiotics. When necessary, a plasmid with appropriate antibiotic resistance was introduced into the donor or recipient strains to facilitate screening.

### Statistical analysis

Statistical analyses were performed using GraphPad Prism Software (GraphPad Prism 8.01). All experiments were performed in at least three independent replicates. Statistical analyses in mice were analyzed using the Mann–Whitney test. All other experiments were analyzed using unpaired, two-tailed Student’s *t*-test. Error bars represent ±SD. ^*^P < .05; ^*^^*^P < .01; ^*^^*^^*^P < .001.

The following assays were described in Supplementary Methods:

- Plasmids construction
- In-frame deletion and complementation
- Overexpression and purification of recombination proteins
- Protein toxicity assay
- Isothermal titration calorimetry (ITC)
- Electrophoretic mobility shift assay (EMSA)
- Glutathione *S*-transferase (GST) pull-down assay
- Bacterial two-hybrid assay
- Growth inhibition assay
- Protein secretion assay
- Western blot analysis
- Construction of chromosomal fusion reporter strains and β-galactosidase assay
- Quantitative real-time PCR (qRT–PCR)
- Metal reconstitution assay
- Ferene S staining assay
- Fluorophore labeling of proteins
- Fluorescent labeling of live bacteria
- Flow cytometry analysis
- Murine infection and competition assays *in vivo*
- Microbiota analysis

## Results

### TepC mediates exploitative competition by acting as a proteinaceous siderophore

**Figure 1:**
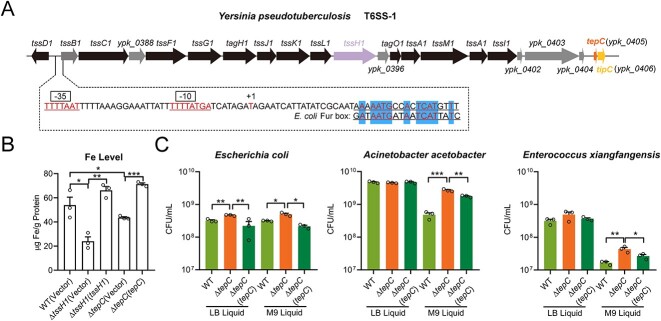
The iron-binding T6SS effector TepC mediates exploitative competition. (A) Identification of the Fur-binding site in the promoter region of the T6SS-1 gene cluster. The putative Fur-binding site was identified by the online software Softberry and was indicated by shading. The −35 and −10 elements of the T6SS-1 promoter were boxed. +1 denotes the transcription start point. (B) T6SS-1 and TepC are involved in iron acquisition. Stationary-phase *Yptb* strains grown in the M9 medium were washed twice. Iron associated with bacterial cells was measured by ICP-MS. (C) TepC is involved in exploitative competition. Interspecies competition assays between indicated *Yptb* donor strains and recipient strains like *Escherichia coli*, *Acinetobacter acetobacter*, and *E. xiangfangensis* in LB or M9 liquid medium, respectively. Donor and recipient strains were mixed 1:1 and then grown for 24 h at 30°C. The CFU of *E. coli*, *A. acetobacter*, or *E. xiangfangensis* was measured based on plate counts. Data are mean ± SD from three biological replicates. ^*^, *P* < .05; ^*^^*^, *P* < .01; ^*^^*^^*^, *P* < .001.

Analysis of the T6SS-1 promoter of *Yptb* revealed a putative binding site for the ferric uptake regulator (Fur) (Fig. 1A), the main transcriptional regulator involved in iron homeostasis in bacteria [39]. Incubation of the biotin-labeled T6SS-1 promoter probe with His_6_-Fur led to hindered mobility of the probe in EMSAs, and this was abolished by the addition of excess unlabeled promoter probe but not promoter probe containing substitution mutations in the Fur-binding site (Fig. S1A), indicating a specific interaction. Consistent with the finding that Fur specifically interacts with the T6SS-1 promoter, deletion of *fur* markedly enhanced the activity of the *P_T6SS1_::lacZ* reporter fusion and expression of the representative T6SS-1 genes (Fig. S1B and C). The negative regulation of T6SS1 by Fur suggests its role in iron acquisition. Furthermore, the expression of T6SS-1 genes was induced in iron-limiting conditions (Fig. S1D). As predicted, deletion of *tssH1* (also known as *clpV1*) strongly reduced the intracellular Fe content, and this defect was restored to wild-type (WT) level by complementation, demonstrating that T6SS-1 is involved in iron acquisition (Fig. 1B).

T6SS is reportedly involved in transporting metal ions via the secretion of proteinaceous metallophores, such as zincophores and manganeseophores [37, 38]. To investigate whether the *Yptb* T6SS-1 may also secrete proteinaceous siderophores to acquire iron, the T6SS-1 gene cluster was analyzed for putative effectors with iron-binding activity (Fig. 1A). Downstream of the T6SS-1 gene cluster, we identified an 81-residue hypothetical protein (YPK_0405, hereafter, TepC). Although the secretion of TepC was readily detected in WT supernatants, TepC levels were greatly reduced in Δ*tssH1*, and the secretion defect was restored to WT levels by complementation, demonstrating that TepC is a T6SS-1 effector (Fig. S2A and B). In addition, ITC and atomic absorption spectrometry analysis showed that TepC can specifically bind Fe^3+^ and Mg^2+^ but not Zn^2+^, Ca^2+^, Mn^2+^, or Fe^2+^ (Fig. S2C–H and J). The iron-specific Ferene S staining assay indicated that only iron-bound holo-TepC turned blue, whereas the iron-free apo form of TepC remained colorless, confirming that TepC binds to Fe^3+^ but not Fe^2+^ (Fig. S2I).

The finding that T6SS-1 secretes a Fe^3+^-binding effector prompted us to investigate the role of TepC in iron acquisition. Compared to the WT strain, the Δ*tepC* mutant exhibited a clear decrease in intracellular iron concentration, further corroborating the involvement of TepC in iron uptake (Fig. 1B). Although the Δ*tepC* mutant exhibited a similar growth rate to that of the WT strain in nutrient-rich YLB, its growth was severely impaired (relative to WT) in an iron-depleted medium containing the iron chelator ethylenediamine-*N*,*N*′-bis 2-hydroxyphenylacetic acid. However, the growth defect of the Δ*tepC* mutant in the iron-depleted medium could be restored by complementation of the *tepC* gene or supplementation with excess Fe^3+^ (Fig. S2K–M).

In addition to mediating interference competition, some T6SSs reportedly conferred a competitive advantage on bacteria by enhancing their capacity to obtain micronutrients for exploitative competition [37-39]. Deletion of *tepC* reduced the interspecies competitive strength of *Yptb* against Gram-negative *E. coli*, *Acinetobacter acetobacter*, and Gram-positive *E. xiangfangensis* in iron-limited M9 liquid medium. By contrast, in the iron-rich LB medium, the Δ*tepC* mutant displayed similar competitive strength to WT or the complemented Δ*tepC*(*tepC*) strain against *A. acetobacter* and *E. xiangfangensis* (Fig. 1C). Unexpectedly, the Δ*tepC* mutant still exhibited reduced competitive strength against *E. coli* in the iron-rich medium (Fig. 1C), suggesting other unknown mechanisms are involved in TepC-mediated interbacterial competition with *E. coli*.

### TepC mediates interference competition by acting as a nuclease toxin

**Figure 2:**
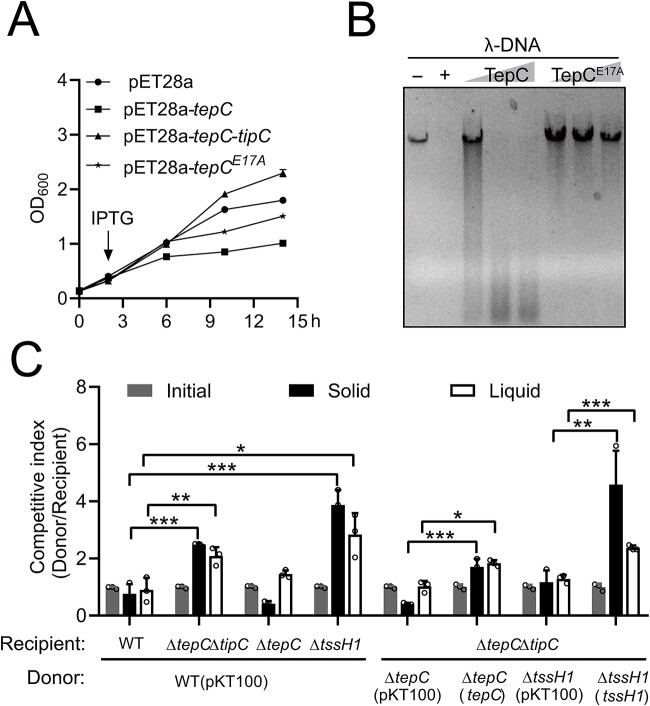
TepC mediates contact-independent interference competition as a DNase toxin. (A) TepC is toxic to *E. coli*. Growth curves of *E. coli* BL21(DE3) containing indicated plasmids were determined by measuring OD_600_ from 0 h to 14 h. (B) DNase assays indicating the integrity of λ-DNA coincubated without (−) or with the DNase I control (+), TepC (0.25, 0.5, 1 μM), or TepC^E17A^ (0.25, 0.5, 1 μM) at 37°C for 30 min *in vitro*. Reaction products were analyzed using agarose gel electrophoresis. (C) Intraspecies competition between the indicated *Yptb* donor and recipient strains in the M9 medium. An equal amount of donor and recipient strains were mixed and grown on a solid or liquid medium for 24 h at 30°C. The competitive index of the donor and recipient strains was calculated based on plate counts. Data are mean ± SD from three biological replicates. ^*^, *P* < .05; ^*^^*^, *P* < .01; ^*^^*^^*^, *P* < .001.

The expression of TepC in *E. coli* leads to significant growth suppression, suggesting that TepC is cytotoxic to *E. coli* cells (Fig. 2A). This growth inhibition was alleviated by the coexpression of the downstream YPK_0406 gene (hereafter, TipC), which forms a bicistron with TepC. A direct interaction between TepC and TipC was revealed in GST pull-down assays (Fig. S3A), and a binding *Kd* of 0.012 ± 0.007 μM was obtained by ITC analysis (Fig. S3B). These findings suggest that, in addition to its iron binding activity, TepC also functions as a canonical T6SS toxin, whereas TipC functions as its immunity protein.

The TipC immunity protein contains an Imm26 domain, as annotated by KEGG SSDB Motif Search (https://www.kegg.jp/ssdb-bin/ssdb_motif?kid=ypy:YPK_0406), and proteins carrying this domain are usually present in bacterial polymorphic toxin systems and encoded by genes that are closely linked to genes encoding the toxin, which usually contains a nuclease domain belonging to the Tox-URI1 or Tox-HNH family [52]. No predictable functional domain could be identified in TepC using the BLASTP search or other bioinformatics tools. The Mg^2+^-binding capacity of TepC suggests its nuclease activity (Fig. S2D). Incubation of purified TepC with λ-DNA led to DNA degradation (Fig. 2B). Likewise, TepC degraded the circular plasmid pUC19, demonstrating that it is an endonuclease (Fig. S3C). The DNase activity of TepC depends on the presence of Mg^2+^ but not Fe^3+^ (Fig. S3D). The ClustalX 2.1-based predictions suggested that the conserved E17 residue may be crucial for DNase activity (Fig. S3E). As predicted, the mutation of E17 substantially attenuated DNase activity and toxicity to *E. coli* (Fig. 2A and B and Fig. S3C). These results demonstrate that TepC is a nuclease toxin with DNase activity and that E17 is the key residue for degrading DNA.

To investigate whether the TepC toxin participates in bacterial antagonism, a classic function of antibacterial T6SS effectors [34, 46], we performed both intraspecies and interspecies competition assays. *Yptb* WT exhibited increased growth advantage over the Δ*tepC*Δ*tipC* recipient under both contact-dependent and contact-independent conditions, and this advantage was abrogated by the deletion of *tepC* from the donor or by the presence of *tipC* in the recipient (Fig. 2C and Fig. S3F). The *Yptb* WT also exhibited a stronger competitive advantage against the Δ*tssH1* recipient, as compared with Δ*tepC*Δ*tipC* recipient, especially on solid media (Fig. 2C and Fig. S3F). This result implies the existence of additional T6SS-1 effectors that enhance the competitiveness of the donor in a contact-dependent manner. Similarly, the deletion of *tepC* from the *Yptb* WT donor strongly abrogated its competitive advantage over the *E. coli* recipient in interspecies competition assays. The competitive disadvantage of Δ*tepC* was rescued by complementation with *tepC* but not with the catalytically inactive *tepC ^E17A^* allele (Fig. S3G).

Because TepC can mediate contact-independent competition, it must enter target cells independently of the T6SS needle. As predicted, the addition of purified TepC to the liquid medium reduced the survival of *E. coli* (Fig. S3H). This indicates that secreted TepC possesses an intrinsic cell entry mechanism that allows it to enter target cells to access its DNA target. Together, these findings demonstrate that TepC, a proteinaceous siderophore with nuclease activity, participates in both interference competition and exploitative competition.

### TepC engages the outer membrane receptor TdsR for cell entry

**Figure 3:**
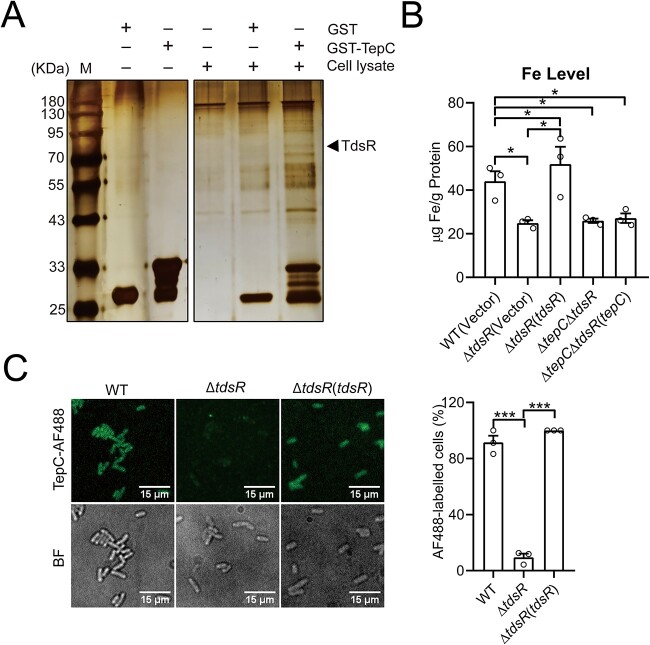
TepC engages TdsR for target cell entry. (A) Identification of TdsR as the outer membrane receptor of TepC. GST•Bind beads coated with GST-TepC or GST were incubated with *Yptb* cell lysates. After the elimination of unbound proteins by washing, retained proteins resolved by SDS-PAGE were visualized with silver staining and specific protein bands were identified with mass spectrometry. (B) TdsR contributes to iron acquisition. Stationary-phase *Yptb* strains were collected and washed with M9. Iron associated with bacterial cells was measured by ICP-MS. (C) Fluorescence labeling of relevant *Yptb* strains with TepC-AF488 (left) and their corresponding quantifications of protein entry (right). Scale bars: 15 μm. Data are mean ± SD from three biological replicates. ^*^, *P* < .05; ^*^^*^^*^, *P* < .001.

All contact-independent T6SS toxins and metallophores require outer membrane receptors to enter target cells [37, 38, 46]. To identify the putative receptors that mediate TepC cell entry, we performed a GST pull-down screen by incubating GST-TepC–coated beads with *Yptb* cell lysates. Mass spectrometry identified an 80-kDa TonB-dependent siderophore Receptor protein (YPK_0815; hereafter, TdsR) that was specifically retained by GST-TepC (Fig. 3A). The interaction between TdsR and TepC was confirmed using a bacterial two-hybrid assay (Fig. S4A). Direct binding of TepC-GFP to the cell surface of *Yptb* cells was observed via fluorescence microscopy, which revealed that most of the *Yptb* WT and the Δ*tdsR*(*tdsR*) complemented cells, but only 18.7% of the Δ*tdsR* mutant cells, were labeled with green fluorescence (Fig. S4B). Furthermore, deletion of *tdsR* significantly reduced intracellular iron accumulation, which corroborates that the protein acts as a TonB-dependent siderophore receptor. However, although complementation of the *tepC* gene in Δ*tepC* completely restored intracellular iron concentrations to WT levels, it had no effect in the *ΔtepC*Δ*tdsR* mutant, which supports the assertion that the iron transport activity of TepC is mediated by TdsR (Fig. 3B).

The TonB-dependent siderophore receptors also transport microcins and bacteriocins through the outer membrane of target bacteria [53-55]. The direct binding between TepC and TdsR prompted us to investigate whether TdsR serves as a transporter for mediating TepC cell entry. Therefore, we performed fluorescence-based assays using Alexa Fluor 488-conjugated TepC to investigate protein import. As predicted, AF488-conjugated TepC could enter the *Yptb* and *E. coli* WT cells but not Δ*tdsR* cells, suggesting that TepC hijacks the outer membrane receptor TdsR for cell entry (Fig. 3C and Fig. S4C). Consistently, the *E. coli* Δ*tdsR* mutant resists TepC treatment, because it is unable to take up the toxin (Fig. S4D).

To further determine the role of TdsR in TepC-mediated contact-independent T6SS antagonism, we performed intraspecies and interspecies competition assays. Although the *Yptb* WT donor had a TepC-dependent competitive advantage over *Yptb* Δ*tepC*Δ*tipC* and the *E. coli* WT recipient, this advantage was diminished when the *Yptb* WT donor was cocultured with *Yptb* Δ*tepC*Δ*tipC*Δ*tdsR* and the *E. coli* Δ*tdsR* recipient in liquid M9 medium (Fig. S4E and F). These results indicate that TepC adopts a microcinlike cell entry mechanism to mediate both interference competition and exploitative competition. However, the *Yptb* WT strain still exhibits substantial competitive advantages over the Δ*tepC*Δ*tipC*Δ*tdsR* strain under contact-dependent conditions on solid medium (Fig. S4E). This suggests that while TdsR is necessary for contact-independent TepC killing, it is not required for contact-dependent TepC killing.

### Fe^3+^ promotes the cell entry of TepC

**Figure 4:**
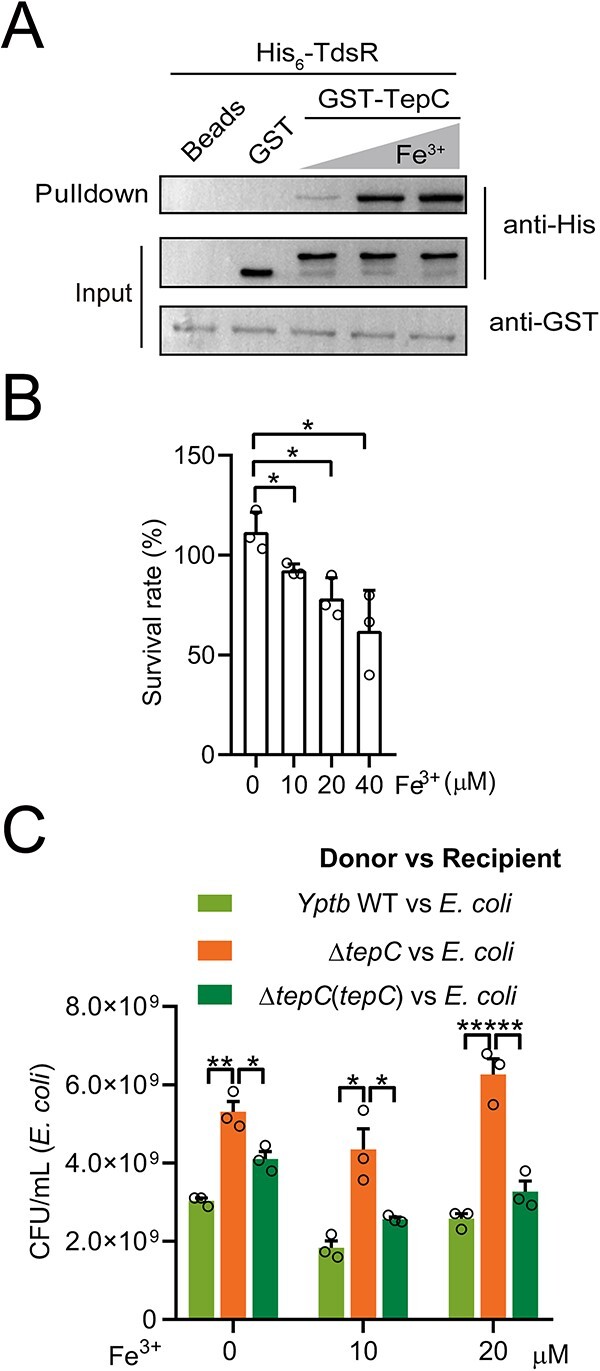
Fe^3+^ facilitates the binding and cellular uptake of TepC. (A) Direct binding between TepC and TdsR in increasing concentrations of Fe^3+^ was examined by GST pull-down assays. (B) Effects of Fe^3+^ on the toxicity of TepC protein to *E. coli* cells. Bacterial cells were diluted 40 times in M9 medium with increasing concentrations of Fe^3+^ (0 to 40 μM) and then treated with TepC protein (0.1 mg ml^−1^) for 1 h. The survival rates of bacterial cells were determined by counting the CFUs after treatment. (C) Effects of Fe^3+^ on TepC-mediated interspecies competition. Indicated *Yptb* donor and *E. coli* recipient strains were mixed 1:1 in M9 liquid containing increasing concentrations of Fe^3+^ (0 to 20 μM) and then grown for 24 h at 30°C. The CFU of recipient strains was measured based on plate counts. Data are mean ± SD from three biological replicates. ^*^, *P* < .05; ^*^^*^, *P* < .01.

Because TepC is a bifunctional effector with both toxic and siderophore properties, we investigated the impact of Fe^3+^ binding on TepC cellular uptake. The interaction between TdsR and TepC required the presence of Fe^3+^ as evidenced by GST pull-down assays (Fig. 4A). When TepC-GFP proteins were incubated with *E. coli* cells and increasing concentrations of Fe^3+^, the amounts of GFP-labeled cells detected by flow cytometry analysis increased (Fig. S5A). The binding of TepC-GFP to the *E. coli* cell surface depends on the presence of the TdsR receptor, because deletion of *tdsR* decreased the quantity of GFP-labeled cells from 33.1% to 13.6%, but GFP-labeled cells were restored to WT levels by complementation. The effect of Fe^3+^ on the binding of TepC-GFP to *E. coli* is specific because similar quantities of Ca^2+^ and Mn^2+^ did not enhance the amounts of GFP-labeled cells (Fig. S5B).

The finding that Fe^3+^ favored the TdsR-TepC interaction suggests that Fe^3+^ promotes the cell entry of TepC. To test this hypothesis, we investigated the protein import of AF488-conjugated TepC in the presence and absence of Fe^3+^. *E. coli* cells incubated with TepC-AF488 in the presence of Fe^3+^ yielded fluorescent bacteria, but those incubated in the absence of Fe^3+^ were only weakly labeled (Fig. S5C), indicating that Fe^3+^ is important for the binding of TepC to cells. Similarly, Fe^3+^ boosted the inhibitory activity of exogenously supplemented TepC against *E. coli*, because increasing concentrations of Fe^3+^ decreased the survival of *E. coli* in the M9 medium (Fig. 4B). Moreover, the *Yptb* WT exhibited a considerable competitive advantage over *E. coli* in the presence of Fe^3+^ (Fig. 4C). These results demonstrate that Fe^3+^ facilitates binding between TepC and the outer membrane receptor TdsR, resulting in the subsequent entry of TepC into bacterial cells.

### TepC punishes cheaters by using DNase activity to privatize iron

**Figure 5:**
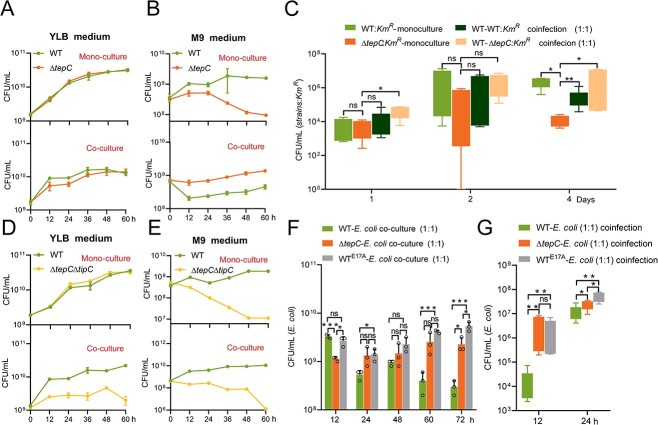
TepC punishes cheaters who exploit it. (A, B, and D, E) Kinetic curves of relevant *Yptb* strains by counting CFUs of each strain at multiple time intervals. *Yptb* WT, Δ*tepC,* or Δ*tepC*Δ*tipC* strains were cultured individually in the YLB (A, D upper) or M9 medium (B, E upper) or cocultured in a 1:1 pairwise mixture in the YLB (A, D lower) or M9 medium (B, E lower). To facilitate CFU counting, *Yptb* WT and mutant strains were labeled with plasmids with different antibiotic resistance. (C) Individual infection of mice with *Yptb* WT (WT*:Km^R^*-monoculture) or Δ*tepC* (Δ*tepC:Km^R^*-monoculture), or coinfection with WT and WT (WT-WT*:Km^R^* coinfection), or WT and Δ*tepC* (WT-Δ*tepC:Km^R^* coinfection) with the same number of each bacteria (5 × 10^8^ CFU). The CFUs of Km^R^ strains in the cecum were counted at the indicated time points. (F) *In vitro* cheating and policing experiments between indicated *Yptb* strains (cooperator) and *E. coli* (cheater) in iron-limited conditions. The CFUs of *E. coli* were counted on a selective medium at the indicated time points. (G) Coinfection of mice with *E. coli* (cheater) and the same amount of *Yptb* WT, Δ*tepC*, or WT^E17A^ cooperator strain. The CFUs of *E. coli* in the cecum were calculated at the indicated time points. Data in A, B, D, E, and F are mean ± SD from three biological replicates. Data in C and G were compared by using the Mann–Whitney test. ^*^, *P* < .05; ^*^^*^, *P* < .01.

The siderophores are recognized as bacterial public goods that may be exploited by cheaters [14, 15]. To investigate whether the proteinaceous siderophore TepC may be exploited in this way, we measured intraspecific competition between WT and the Δ*tepC* strain. The WT strain was used as a cooperator, whereas the Δ*tepC* strain, which does not produce but acquires TepC under the protection of TipC, was used as a cheater. When cultured separately, the WT strain exhibited the same growth rate as the Δ*tepC* mutant in an iron-rich YLB medium, whereas it grew faster than Δ*tepC* in an iron-limited M9 medium, confirming the role of TepC in facilitating bacterial iron uptake. However, in a mixed coculture, Δ*tepC* outcompeted the WT strain in iron-limited M9 medium (Fig. 5A and B). These observations confirmed the cheating behavior of the Δ*tepC* strain in coculture conditions. Such cheating behavior was critically dependent on the TdsR receptor because the competitive advantage of Δ*tepC* over WT was reduced by deletion of *tdsR* but was restored by complementation (Fig. S6A). Furthermore, infection of groups of mice with either WT or Δ*tepC* strains confirmed that lacking TepC attenuated bacterial replication in the host gut, whereas coinfection with WT substantially enhanced Δ*tepC* replication, demonstrating the successful cheating behavior of the nonproducer Δ*tepC* in mouse gut (Fig. 5C). These observations indicate that the Δ*tepC* mutant acts as a social cheater that exploits TepC.

Because TepC is bifunctional, we postulated that its exploitation may have negative consequences for target cells that lack the immunity protein. In contrast to the Δ*tepC* mutant, the Δ*tepC*Δ*tipC* mutant, which lacked the immunity protein, did not grow better than the WT strain in iron-limited conditions (Fig. 5D and E), unless the immunity gene was complemented (Fig. S6B). We further investigated this by using Δ*tepC*Δ*tipC* and WT^E17A^ (a mutant produced by knock-in of the *tepC^E17A^* allele at the original site on the Δ*tepC* chromosome) as a proof-of-principle strain. However, the Δ*tepC*Δ*tipC* mutant could not be outcompeted by the WT^E17A^ strain, as compared with WT. The WT^E17A^ releases a nontoxic TepC^E17A^ that can be exploited even by a cheater that lacks immunity protein (Fig. S6B and C). These results suggest that TepC possesses a DNase-meditated policing mechanism to punish cheaters.

We tested interspecies competition between *E. coli* (cheater) and each individual *Yptb* strain (cooperator) *in vitro*. As expected, *E. coli* growth was significantly augmented in a coculture with WT^E17A^ under iron-limited conditions, in contrast to its growth in a mixed culture of *E. coli* and *Yptb* WT. This implies that cheating behavior also occurred between *Yptb* and *E. coli*, with the latter being penalized by toxic TepC. *Escherichia coli* growth was weak in the *Yptb* WT–*E. coli* coculture pair, indicating that the negative effects can outweigh the potential benefits of TepC uptake (Fig. 5F). Furthermore, the cheating and punishment of cheaters occurred in the mouse gut, because *E. coli* reaped greater benefits when coinfected with WT^E17A^ than with *Yptb* WT (Fig. 5G). This suggests that both *Yptb* WT and WT^E17A^ can provide benefits to *E. coli*, but WT with toxic TepC can punish cheaters.

As described above, iron-laden TepC may be used as public goods in microbial communities. Moreover, TepC also possesses toxic DNase activity, which enables it to punish nonimmune cheaters and ensure iron privatization. This bifunctionality of the T6SS-secreted effector observed in bacterial competition may be considered a “Trojan Horse mechanism” because the metallophore characteristics of TepC mean it can be used as a public good, but cheaters are punished by its DNase-mediated toxicity.

### TepC facilitates the gut colonization of *Yptb*

**Figure 6:**
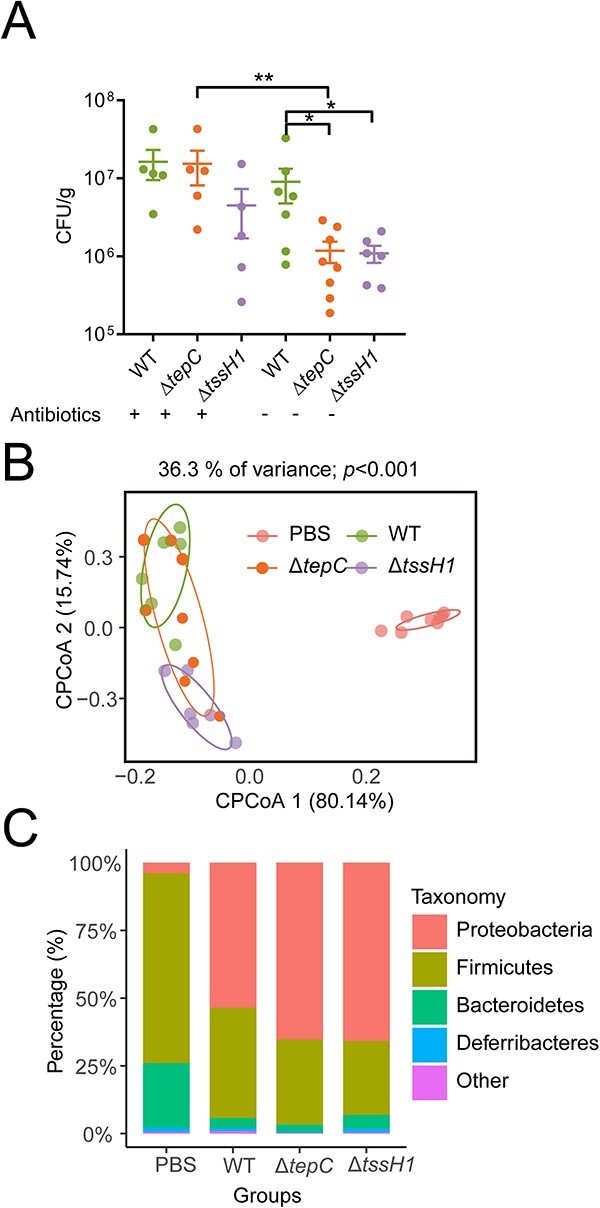
TepC-mediated T6SS antagonism contributes to gut colonization. (A) Mice pretreated with or without antibiotics were orally gavaged with 10^9^ CFUs of different *Yptb* strains (*n* = 5–8), and bacterial loads in the cecum were measured 4 days postinfection. (B, C) The analysis of the 16S rRNA gene amplicon of the cecal contents of experimental mice (*n* > 5 for each group). CPCoA with Bray-Curtis distance showing the beta diversity (*P* < .001, PERMANOVA by Adonis) (B). Phylum-level distribution of native gut microbiota in the four treatments (C). Statistical analysis of experiments in A was carried out using the Mann–Whitney test. ^*^, *P* < .05; ^*^^*^, *P* < .01.

To test whether TepC helps the enteric pathogen *Yptb* to colonize the gastrointestinal tract, antibiotics-treated and untreated mice were gavaged with 10^9^ colony-forming units (CFUs) of *Yptb* WT, Δ*tepC*, or Δ*tssH1* strains. The colonization of each *Yptb* strain in the cecum was determined 96 h after infection. In the absence of antibiotics treatment, there were significantly fewer Δ*tepC* and Δ*tssH1* CFUs than WT CFUs. However, pretreatment with antibiotics greatly reduced the observed differences, and Δ*tepC* even exhibited a similar level of colonization to WT, which might indicate that TepC-mediated T6SS antagonism contributes to *Yptb* colonization by outcompeting gut commensals (Fig. 6A).

Additionally, the V3–V4 regions of 16S rRNA genes from the cecum contents were sequenced to reveal the effects of TepC on gut microbiota during *Yptb* infection. Alpha diversity analysis showed that gut microbiota diversity in *Yptb*-infected mice was significantly reduced compared to a phosphate-buffered saline control group, but Δ*tepC* and Δ*tssH1* had relatively weak effects on the taxonomic diversity of gut microbiota compared to WT, although there were no statistical differences (Fig. S7A). Constrained principal coordinates analysis of the Bray–Curtis distance revealed that the gut microbiota generated by infection with the *Yptb* WT, Δ*tepC*, and Δ*tssH1* strains formed three clusters along the second coordinate axis (Fig. 6B). The WT and Δ*tssH1* groups gathered as separate clusters, whereas the Δ*tepC* groups overlapped with the WT and Δ*tssH1* groups. These results might indicate that both Δ*tepC* and Δ*tssH1* influence the overall composition of gut microbiota, and the effect of Δ*tepC* on gut microbiota was less than that of Δ*tssH1*, implying other T6SS-1 secreted effectors are also involved. We also found that *Yptb* was more abundant in the WT group than in the Δ*tepC* and Δ*tssH1* groups (Fig. S7B), consistent with the results of the colonization experiments.

After infection with *Yptb*, the relative abundance of the native gut microbiota in mice changed considerably with regard to *Proteobacteria*, *Firmicutes*, and *Bacteroidetes* (Fig. 6C). To gain further insight into the role of TepC in gut colonization, we examined differences in the gut microbiota of the WT and Δ*tepC* groups at the amplicon sequence variants (ASVs) level (Fig. S7C). Compared to the WT group, ASVs enriched in the Δ*tepC* group were from the Firmicutes and Proteobacteria. Comparisons at the genus level revealed that the relative abundances of *Enterococcus*, *Escherichia/Shigella*, *Acetatifactor*, *Flavonifractor*, *Terrisporobacter*, *Propionibacterium*, *Anaerotruncus*, and *Clostridium* had significantly increased in the Δ*tepC* group, which implies that TepC provides *Yptb* with a competitive advantage over a broad range of Gram-negative and Gram-positive bacteria (Fig. S7D). To investigate this hypothesis, we performed *in vivo* competition assays. Mice pretreated with antibiotics were inoculated with *E. coli*, *Salmonella Typhimurium*, or *E. xiangfangensis*, for 24 h before being gavaged with *Yptb* WT or Δ*tepC*. After 36 h of *Yptb* challenge, the intestinal loads of *E. coli*, *S. Typhimurium*, and *E. xiangfangensis* in mice challenged with *Yptb* WT were significantly lower than those of mice challenged with Δ*tepC* (Fig. S7E–G). In contrast, the levels of *Yptb* WT were significantly higher than those of Δ*tepC*. These results could suggest that the TepC-mediated T6SS antagonism pathway plays a crucial role in niche competition by targeting a broad range of gut commensals and enteric pathogens. Furthermore, the TepC protein can only enter cells of some of the target species detected, such as *E. coli* and *S. Typhimurium* (Fig. S8A). For those bacteria without TdsR homologs, TepC could not exert contact-independent antagonism (Fig. 1C). In contrast, TepC could exert contact-dependent antagonism against Gram-negative bacterial cells without TdsR homologs like *P. aeruginosa*, and Gram-positive *S. aureus* and *E. xiangfangensis* competitors, as verified by performing competition assays under conditions promoting cell contact (Fig. S8B).

## Discussion

**Figure 7:**
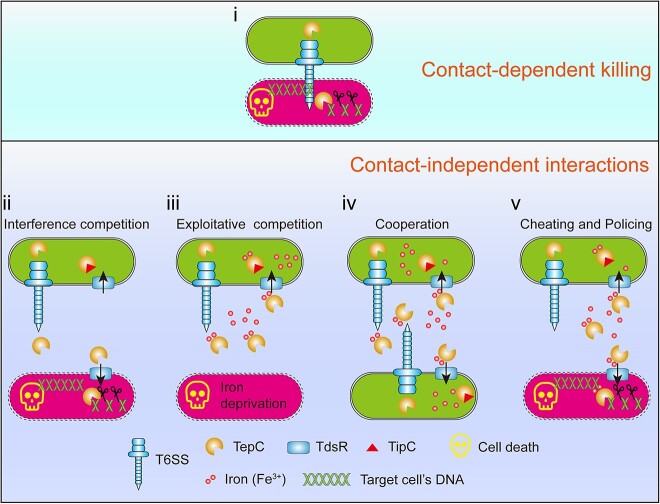
Model of the TepC-mediated cooperation and competition behavior. (i) TepC mediates contact-dependent, receptor-independent interference competition. The toxic TepC produced by *Yptb* cells was contact-dependently, and receptor-independently injected into adjacent nonkin cells to degrade the target cell’s DNA, thus allowing the attacking cells to outcompete nonkin cells to gain growth edges. (ii) TepC mediates contact-independent, receptor-dependent interference competition. TepC is secreted into the extracellular environment by the T6SS, and the secreted TepC can hijack the TdsR receptor on the surface of nonkin cells to enter and degrade genomic DNA. (iii) TepC mediates exploitative competition. In iron-deficient conditions, TepC is secreted into the extracellular environment by the T6SS, which can sequester Fe^3+^ and facilitate their transport into the secreting cell, but restricting nonkin cells to obtain this limited resource. Therefore, the growth of nonkin cells was inhibited. (iv) TepC facilitates cooperation between kin cells. The secreted TepC loaded with Fe^3+^ can be utilized as a public good between kin cells that possess the TdsR receptor and the TipC immunity protein, thus improving the cooperation between kin cells. (v) TepC punishes cheaters that possess the TdsR receptor but not the TipC immunity protein. Under iron-limited conditions, nonkin cells that contain TdsR but not TipC are lured to uptake iron-laden TepC as a public good. However, the uptake of TepC can enable the policing of cheater cells because of its poisonous potential.

Here we identified a versatile T6SS effector, TepC, which mediates both competition and cooperation behaviors in microbial communities and utilizes a lure-to-kill policing mechanism to privatize iron by punishing cheaters (Fig. 7). TepC is a T6SS effector that can mediate both exploitative competition and interference competition. As a bifunctional effector, the toxic TepC is able to load with iron and is used as public goods to facilitate kin-cell cooperation. The iron-loaded TepC hijacks TdsR, an outer membrane TonB-dependent siderophore receptor, to enter target cells. For kin cells, TepC facilitates iron acquisition because its toxic effects are neutralized by the immunity protein TipC. However, TepC kills nonkin cells that lack immunity proteins via its DNase activity, thus providing a simple but effective lure-to-kill policing mechanism to prevent cheater infiltration and enforce cooperation.

The role of TepC in acquiring Fe^3+^ has been verified by several lines of evidence. Firstly, the expression of *Yptb* T6SS-1 and TepC was directly repressed by the ferric uptake regulator Fur (Fig. 1A and Fig. S1C) and induced in iron-limited conditions (Fig. S1D). However, the bacterial killing potential and secretion of TepC in the rich medium indicate its constant expression in such medium, albeit at a low level (Fig. 1C and Fig. S2A). Next, multiple experiments such as ITC and Ferene S staining assay detected the direct binding between TepC and Fe^3+^ but not Fe^2+^ (Fig. S2C and I). Moreover, TepC is necessary for the growth of *Yptb* under Fe^3+^-limited conditions (Fig. S2K–M). Finally, TepC directly interacts with the TonB-dependent siderophore receptor protein TdsR, and this interaction is further promoted by the presence of Fe^3+^ (Figs S4A and S5A). Collectively, these results proved that TepC is a noncanonical proteinaceous siderophore, which is distinct from canonical low-molecular-weight siderophores. Although T6SSs were reported to facilitate micronutrients acquisition by secretion of a panel of proteinaceous metallophores such as zincophores and manganeseophores [37, 38], no T6SS effector that directly binds to iron has been reported. However, our findings reveal that the T6SS-secreted TepC is a dual-functional effector, possessing both toxic DNase and iron uptake capabilities. This dual functionality of TepC provides *Yptb* with competitive advantages in ecological competition.

Ecological competition can be divided into two major types: exploitative competition, which occurs indirectly through the depletion of resources, and interference competition, whereby one individual directly harms another [56, 57]. Specifically, TepC can act as a proteinaceous siderophore, allowing bacteria to gain competitive advantages in exploitative competition, whereas also exerting toxic DNase activity to engage in interference competition. However, the competition effect size of TepC seems to be small, which suggests the possibility that other factors might also contribute to the competitiveness of *Yptb*. Additionally, the variation in expression levels of the T6SS operon and *tepC* in different environments may also contribute to competition outcomes. Nevertheless, TepC could still have important implications in specific ecological contexts or in combination with other factors. This discovery significantly enhances our understanding of the role of T6SS in mediating bacterial competition, as it integrates both exploitative and interference competition within a single effector. Although the evolutionary significance of this dual-function mechanism is still unclear, the presence of TepC endows T6SS with a broader spectrum of antibacterial functions and increases the potential of bacteria to occupy ecological niches.

Social cheating can occur among cooperating groups of bacteria under laboratory conditions [58, 59], but it seldom leads to the collapse of natural populations [60, 61]. Several mechanisms to guard public goods and maintain community stability have been proposed [10, 11, 15, 18-24, 62]. One such mechanism is toxin-based policing, which allows bacteria to selectively target nonkin strains by producing immunity factors along with toxins [8, 11]. The delivery of public goods to kin cells has favorable effects, whereas nonkin cells (which lack immunity against the accompanying toxins) are punished. However, this strategy relies on the joint regulation of toxin-immunity systems with the production of public goods and assumes that cheaters do not produce effective resistance factors [63]. Furthermore, such toxin-based policing mechanisms depend on appropriate environmental dissemination and durability, as well as effective toxins [8]. When the relationship between public goods, toxins, and toxin resistance breaks down, negative selection emerges.

Here, we describe an effective toxin-mediated lure-to-kill policing mechanism that eradicates cheaters using the bifunctional T6SS effector TepC. TepC has both iron-binding capacity and toxic DNase activity, thus combining the public good and toxin function in one molecule. TepC is a beneficial community resource as a siderophore, but its toxic potential enables it to punish cheaters that do not secrete public goods, restricting the proliferation of these cheaters within the community. As a “Trojan horse”–like effector, iron-loaded TepC can even lure cheater cells to uptake the toxin. The convergence of the siderophore activity and toxic potential in one molecule not only improved the efficiency and selectiveness of the policing system but also eliminated the requirement of a complex regulation system to ensure the coupled expression of the toxin and the public good in reported policing systems [8, 10, 11, 25, 64]. This is also a novel mechanism for securing the privatization of iron resources, in which the toxic siderophore TepC can only be used by secreter or kin cells containing the TipC immunity protein, which efficiently prevents nonproducer bacteria from evolving into iron thieves. However, such policing might be vulnerable because the policing effectiveness hangs on the linkage between TepC and TipC expression, and a simple expression of TipC could defect the policing behaviors.

In conclusion, we describe a “Trojan horse”–like T6SS effector, TepC, which mediates both exploitative competition and interference competition (Fig. 7). Its lure-to-kill-based cheater policing mechanism, along with its broad-spectrum antibacterial activity, provides a foundation for novel approaches to treating drug-resistant bacterial infections and addressing imbalances in gut microbiota.

## Supplementary Material

Supplementary_information_1130_wrad028

Supplementary_Table_1_wrad028

Supplementary_Table_2_wrad028

## Data Availability

The 16S rRNA gene sequencing data have been deposited in the National Center for Biotechnology Information GenBank repository (accession numbers: SRR25404125–25404152) and China National Microbiology Data Center (accession numbers: NMDC40041753–40041780). Other data supporting the findings of this study are included in the article and its Supplementary Information files or from the corresponding authors upon request.
