# Supplementary material for: Trojan horselike T6SS effector TepC mediates both interference competition and exploitative competition
Source: ISME J. 2024 Jan 10;18(1):wrad028. doi: 10.1093/ismejo/wrad028 (PMC10833071; doi:10.1093/ismejo/wrad028)
Supplement: Supplementary_information_1130_wrad028 [file supplementary_information_1130_wrad028.docx]

**Supplementary Information for**

**Trojan horse-like T6SS effector TepC mediates both interference competition and exploitative competition**

Li Song^1,2^*, Lei Xu^2^*, Tong Wu^2^*, Zhenkun Shi^2^, Hafiz Abdul Kareem^2^, Zhuo Wang^2^, Qingyun Dai^2^, Chenghao Guo^2^, Junfeng Pan^2^, Mingming Yang^3^, Xiaomeng Wei^1^, Yao Wang^2^, Gehong Wei^1#^, Xihui Shen^2^^#^

^1^State Key Laboratory for Crop Stress Resistance and High-Efficiency Production, College of Natural Resources and Environment, Northwest A&F University, Yangling, Shaanxi, 712100, China.

^2^Shaanxi Key Laboratory of Agricultural and Environmental Microbiology, College of Life Sciences, Northwest A&F University, Yangling, Shaanxi, 712100, China.

^3^College of Plant Protection, Northwest A&F University, Yangling, Shaanxi, 712100, China.

This PDF file includes:

Supplementary Material and Methods

Supplementary Fig. S1 to S8

Supplementary References

**Supplementary Material and Methods**

**Plasmids construction**

Primers used in this study are listed in Supplementary Table 2. To gain plasmid pME6032-*tepC*-*vsvg* to perform secretion assay, primers *tepC*-F-*Eco*RI and *tepC*-R-*Bgl*II were used to amplify the gene *tepC* using the *Yptb* genome as a template. The fragment was digested with restriction endonuclease *Eco*RI and *Bgl*II and inserted into plasmid pME6032 with the same digested sites. The pME6032-*tdsR_EC_* was obtained with primers *tdsR_EC_*-F-*Eco*RI and *tdsR_EC_-*R*-Xho*lI in the same methods. To attain the expression plasmid pET28a-*tepC*, the *tepC* gene was amplified by PCR with primers *tepC-*F*-Eco*RI and *tepC-*R*-Sal*I. Double-digested fragments were inserted into plasmid pET28a with the same restrictive enzyme sites and transformed into *E. coli* TG1 to yield production. Other clones for expression (pET28a-*tipC*, pET28a-*tepC-tipC*, and pGEX6p-1-*tepC*) were achieved with their corresponding primers. As for pET28a-*tepC^E17A^*, QuickMutation™ Plus Site Directed Mutagenesis Kit (Beyotime Biotechnology, catalog no. D0208S) was utilized with primers *tepC^E17A^-*F and *tepC^E17A^-*R. To create the expression plasmid pET28a-*tepC*-*gfp*, the *tepC* fragment without termination codon was amplified using primers *tepC-*F*-Bam*HI and *tepC-*R*-Eco*RI, and the *gfp* fragment was amplified with primers *gfp-*F-*Eco*RI and *gfp-*F-*Sal*I, then both *tepC* and *gfp* fragments were ligated into pET28a to obtain pET28a-*tepC*-*gfp*. For the bacterial two-hybrid experiment, two pairs of primers *tepC-*F*-Xbal*I, *tepC-*R*-Kpn*I and *tdsR-*F*-Bam*HI, *tdsR-*R*-Eco*RI were used to acquire genes *tepC* and *tdsR*. The clones pUT18C-*tepC* and pKT25-*tdsR* were obtained in the same way. To complement the Δ*tepC* and Δ*tepC*Δ*tipC* mutants, primers *tepC*-F-*Bam*HI and *tepC*-R-*Sal*I were employed. The fragment was digested with *Bam*HI/*Sal*I and ligated into plasmid pKT100 to produce pKT100-*tepC*. The complementary plasmids pKT100-*tipC* and pKT100-*tdsR* were similarly constructed. To construct pTargetF1-Δ*tdsR_EC_*, upstream and downstream fragments of *tdsR_EC_* were amplified via PCR with the primer pairs *tdsR_EC_-*up*-*F/*tdsR_EC_-*up*-*R and *tdsR_EC_-*down-F/*tdsR_EC_-*down-R*-Sal*I, respectively. The DNA fragment that codes for *tdsR_EC_* targeted sgRNA was achieved by PCR against pTargetF1 using primer pair *tdsR_EC_-sg20-*F*-spe*I/*tdsR_EC_-sg20-*R. Overlapping PCR was used to combine all three parts with the primer pairs *tdsR_EC_-sg20-*F*-spe*I/*tdsR_EC_-*down-R*-sal*I and the resulting fragment was ligated into pTargetF1 pre-digested with *Spe*I/*Sal*I using Gibson Assembly. To obtain pDM4-Δ*tepC*Δ*tipC* derivatives, the upstream and downstream fragments were acquired with primer pairs *tepC-*M1F*-Bgl*II/*tepC-*M1R, and *tipC-*M2F/*tipC-*M2R*-Sal*I, respectively, connected together by overlap PCR, and then ligated with plasmid pDM4. pDM4-Δ*tdsR*, pDM4-*tepC* and pDM4-*tepC^E17A^* were obtained with the same method. All constructs were validated by DNA sequencing.

**In-frame deletion and complementation**

In-frame deletion mutants and knock-in strains were generated as described [1]. Briefly, the plasmid pDM4-Δ*tepC* was transferred into *Yptb* from *E. coli* S17-1 by conjugation. Next, the *Yptb* obtaining plasmid successfully was screened using YLB plates (with chloramphenicol, 20 μg·ml^-1^) and grown on suitable YLB liquid until OD_600_ to 2.4. A selective plate (YLB agar containing 20% sucrose) was used to force *Yersinia* to lose pDM4. Finally, PCR was performed to screen the correct mutant. Δ*tdsR* and Δ*tepC*Δ*tipC* were produced similarly. The plasmid pDM4-*tepC^E17A^* was used to knock in the *tepC^E17A^* allele into the original site in Δ*tepC* to produce the WT^E17A^ strain with the same method. The pKT100-*tepC*, pKT100-*tdsR* and pKT100-*tipC* plasmid were electroporated into corresponding mutant strains for complementation. In addition, the CRISPR-Cas9 system (Clustered regularly interspaced short palindromic repeats with Cas9) was used to delete the gene *tdsR_EC_* in *E. coli* [2, 3]. Briefly, plasmid pTargetF1-Δ*tdsR_EC_* was electroporated into *E. coli* DH5α pre-transferred with the vector pCas at 30°C. A selective medium (LB containing 50 μg ml^-1^ kanamycin and 20 μg ml^-1^ chloramphenicol) was used to screen the mutant colonies. The plasmids pTargetF and pCas were successively eliminated by adding 1 mM IPTG induction at 30°C and overnight incubation at 37°C, respectively. The *E. coli* Δ*tdsR_EC_* deletion mutant was trans-complemented with pME6032 carrying the corresponding gene.

**Overexpression and purification of recombination proteins**

Bacteria harboring expression plasmid pET28a-*tepC*, pET28a-*tipC*, and pGEX6P-1-*tepC* were grown in LB liquid at 37°C respectively, then re-inoculated in fresh LB medium with the ratio of 1:100 until OD_600_ to 0.6. 0.2 mM isopropyl β-D-1-thiogalactopyranoside (IPTG) was added to induce the expression of the target protein at 16°C for about 18 h. Cells were collected and disrupted using sonification. The target protein was purified with the His•Bind Ni-NTA resin or GST•Bind Resin (Novagen, Madison, WI). Purified proteins were dialyzed in PBS buffer or Tris-HCI buffer according to experimental requirements. In order to purify the outer membrane receptor TdsR, cell pellets were obtained using the same way mentioned above. Then, cells were resuspended with buffer (20 mM Tris-HCl, pH 8.0, 100 mM glycine, 6 M urea) and disrupted with sonification. After collecting the supernatant, the recombinant TdsR protein was purified with the His•Bind Ni-NTA resin and renatured at 4°C overnight in refolding buffer (55 mM Tris-HCl, 0.21 mM NaCl, 0.88 mM KCl, 880 mM L-arginine, 0.5% SB-12, pH = 7.0). Renatured TdsR protein was dialyzed in a specific dialysis buffer (55 mM Tris·HCl, 0.21 mM NaCl, 10 mM L-arginine, and 0.5% SB-12, pH 6.5).

**Protein toxicity assay**

Stationary phase bacteria cells were collected, washed and diluted 40-fold into M9 medium, and treated with purified TepC protein (0.1 mg ml^–1^) at 30°C with shaking at 100 rpm for 60 min. After treatment, the cultures were serially diluted and plated onto agar plates, and colonies were counted after 36 h growth at 30°C. Percentage survival was calculated by dividing the number of CFU of treated cells by the number of CFU of cells without toxin treatment. All these assays were carried out in triplicate at least three times [4].

**Isothermal titration calorimetry (ITC)**

Protein binding affinity was determined using isothermal titration calorimetry (ITC) at 25°C with a NANO-ITC 2G microcalorimeter (TA Instruments, New Castle, DE, USA). For examining TepC-TipC and TepC-λ DNA interaction, both samples were diluted into PBS buffer. Titrations were carried out with TepC in the syringe and TipC or λ DNA in the cell. As for metal ions and TepC interaction, titrations were carried out with metal ions in the syringe and TepC in the cell. Each titration experiment consisted of 25 injections with 300-s intervals between each injection. The ITC data were analyzed with the Nano Analyze software (TA Instruments, USA) and fitted using a single-site binding model. All ITC experiments were performed at least three times [4-6].

**Electrophoretic mobility shift assay (EMSA).**

Electrophoretic mobility shift assay was performed using biotin 5’-end-labeled promoter probes as described [7]. The biotin-labeled BioT6SS1-p probe and the unlabeled T6SS1-p and T6SS1-pM competitor DNA were obtained by annealing primer pairs BioT6SS-1pF */* T6SS-1pR, T6SS-1pF */* T6SS-1pR, and T6SS-1pMF */* T6SS-1pMR, respectively (Supplementary Table 2). Each EMSA reaction (20 µl) was prepared according to the manufacturer’s protocol (LightShift Chemiluminescent EMSA kit; Thermo Fisher Scientific): 1×binding buffer, 50 ng poly (dI-dC), 2.5% glycerol, 0.05% NP-40, 5 mM MgCl_2_, 20 fmol Biotin-DNA, 4 pmol unlabeled DNA as competitor and different concentrations of recombinant Fur protein. After incubation at 25°C for 20 min, aliquots of binding reaction mixtures were separated by 6% polyacrylamide native gel and transferred to a Biodyne B Nylon membrane (Thermo Fisher Scientific). Migration of biotin-labeled probes was detected by streptavidin-horseradish peroxidase conjugates that bind to biotin and chemiluminescent substrate according to the manufacturer’s protocol.

**GST pull-down assay**

GST pull-down assay was performed as described with minor modifications [8]. To examine the interaction between TepC and TipC, purified GST, GST-TepC was incubated with pre-washed glutathione beads for 2 h. After washing the excess-unbounded GST-tagged protein, His_6_-TipC was added equally per sample at 4°C overnight. A 5% mixture was acquired in each sample as input. Beads were washed with GST binding buffer containing 300 mM NaCl, 500 mM NaCl, and 700 mM NaCl, respectively for 3 times. Each sample was collected and tested by SDS-PAGE and western blot analysis. The interaction between TepC and TdsR was performed in the same manner. To screen for the outer membrane receptors that interacted with TepC, GST and GST-TepC were incubated with pre-washed glutathione beads for 2 h. Then, *Yptb* cell lysates were mixed with per sample equally at 4°C overnight. After washing in gradient elution buffer with different NaCl concentrations, the captured protein was resolved using SDS-PAGE and visualized by silver staining (Bio-Rad).

**Bacterial two-hybrid assay**

Bacterial two-hybrid complementation assays were performed as described previously [9]. Briefly, pKT25-*tdsR* and pUT18C-*tepC* were co-transformed into *E. coli* BTH101 and grown on MacConkey plates containing ampicillin (100 μg ml^-1^), kanamycin (50 μg ml^-1^) and IPTG (1 mM) at 30°C. pKT25 + pUT18C were served as negative control and pKT25-*zip* + pUT18C-*zip* were served as positive control. Interactions were visualized with the MacConkey maltose plates and quantified with β-galactosidase assay.

**Growth inhibition assay**

*E. coli* BL21(DE3) containing plasmid pET28a, pET28a-*tepC*, pET28a-*tepC*-*tipC* or pET28a-*tepC^E17A^* were grown in LB medium to stationary phase. After diluting 100-fold into fresh LB medium for 2 h, 0.3 mM IPTG was added and OD_600_ was measured from 0 h to 14 h [4].

**Protein secretion assay**

Protein secretion assays were performed as previously described [4, 5]. Briefly, overnight grown *Yptb* strains harboring pME6032-*tepC*-*vsvg* were transferred into 300 ml YLB medium and induced with 1 mM IPTG until OD_600_ reached 0.60-0.65. 2 mL culture was collected and the pellets were resuspended in an SDS-PAGE sample buffer to serve as the total pellet. A total volume of 250 ml culture was centrifuged at a speed of 5000 rpm for 20 min, and the resulting supernatant was subjected to an additional centrifugation step at a speed of 9900 rpm for 50 min. The ultimate supernatant was collected and subjected to filtration using a 0.22 μm pore size filter (Millipore, MA). The proteins were collected by filtration using a nitrocellulose filter (BA85, Whatman, Germany) three times. Protein on the filter was dissolved in 100 μl of SDS sample buffer for 15 min at 65°C, and then boiled for 10 min to facilitate the recovery of the protein. Protein samples of both total pellets and culture supernatants were resolved by SDS-PAGE and detected by western blotting. All samples were normalized to the OD_600_ of the culture and volume used in the preparation.

**Western blot analysis.**

Protein samples resolved using SDS-PAGE were transferred onto PVDF membranes (Millipore, MA). After blocking with 5% BSA, specific primary antibodies were incubated at 4°C overnight, which included: anti-VSVG (Santa Cruz Biotechnology, catalog no. sc-365019, lot number: B0916), 1:1000; anti-RNAP (Santa Cruz Biotechnology, catalog no. sc-56766, lot number: F2514), anti-GST (Santa Cruz Biotechnology, catalog no. sc-53909, lot number: F2413), and anti-His (Santa Cruz Biotechnology, catalog no. sc-8036, lot number: I1018), 1:500. Membrane was washed with TBST buffer (50 mM Tris-HCl, 150 mM NaCl, 0.05% Tween 20, pH 7.4) for 5 times, and then incubated with secondary antibodies (Shanghai Genomics, catalog no. DY60203, lot number: 20614) at 4°C for 4 h. After washing with TBST, signals were visualized by ECL plus kit (GE Healthcare, Piscataway, NJ) with a Chemiluminescence Imager (Tanon 5200Multi, Beijing).

**Construction of chromosomal fusion reporter strains and β-Galactosidase assay**

The *lacZ* fusion reporter vector pDM4-*T6SS-1p::lacZ* was transformed into *E. coli* S17-1λpir and mated with relevant *Yptb* strains according to procedures described previously. The *lacZ* fusion reporter strains were grown in YLB broth at 30°C and β-galactosidase activity was assayed with ONPG as the substrate [4].

**Quantitative Real-time PCR (qRT-PCR).**

Total RNA was extracted using the RNAprep Pure Cell/Bacteria Kit (TIANGEN, Beijing, China) along with the DNase I Kit (Sigma-Aldrich, Taufkirchen, Germany). RNA concentration was measured with NanoDrop 2000 (Thermo Fisher Scientific, USA). The TransStart Green qPCR Super-Mix (TransGen Biotech, Beijing, China) and the Bio-Rad CFX96 Real-Time PCR Detection System (Bio-Rad, USA) were utilized to determine mRNA abundance according to the manufacturer’s instructions. Primers used in this study are listed in Supplementary Table 2. The relative abundance of 16S rRNA was used as an internal standard to normalize the results.

**Metal reconstitution assay**

The metal reconstitution assay was performed to determine the iron binding activity of TepC as described [1, 6, 10]. Briefly, purified TepC protein was added to a solution (25 mM Tris, 25 mM diethylene triamine pentaacetic acid, 10% glycerol, pH 7.5) for 1 h to remove metal ions bound to the protein, and then dialyzed in buffer (25 mM Tris, 10% glycerol, pH 7.5) at 4 °C. For reconstitution with metal ions, the resulting TepC protein (10 µM) was incubated with metal ions (Fe^3+^, Mg^2+^, Zn^2+^ and Mn^2+^, 25 µM) for 30 min on ice. These solutions were dialyzed again in buffer (25 mM Tris, 10% glycerol, pH 7.5) to remove unbound metal ions and the metal ions bound to the protein were measured with atomic absorption spectroscopy (ZEEnit 650P; Analytik Jena, Jena, Germany).

**Ferene S staining assay**

TepC was also tested for iron binding with a Ferene-S staining assay performed as described with minor modifications [11]. Briefly, purified protein TepC was dialyzed overnight at 4 °C against 200 μM EDDHA to remove iron and then dialyzed in a buffer (25 mM Tris, 10% glycerol, pH 7.5) to remove EDDHA. The apo-TepC was reconstitute in different concentrations of Fe^3+^ for 2 h at 4 °C. After removing unbound Fe^3+^ by dialysis, apo-TepC and reconstituted TepC were combined with the Ferene S stain (0.75 mM Ferene S, 2% acetic acid) in the presence or absence of 15 mM thioglycolic acid, and dot blotted onto a nitrocellulose membrane to observe for the blue color.

**Mass spectrometry**

Mass spectrometry to identify putative receptors was performed as previously described [4]. The individual protein bands on the gel were excised, which were then subjected to trypsin digestion and analyzed by matrix-assisted laser desorption/ionization/mass spectrometry (Voyager-DE STR, Applied Biosystems, Waltham, MA).

**Fluorophore labeling of proteins**

Fluorophore labeling of proteins was performed as previously described [12]. Briefly, TepC pre-treated with 5 mM DTT was incubated with 10 mM maleimide fluorophores (Thermo Fisher Scientific, USA, catalog no. A10254) at 4°C in the dark for 8-10 h. 2 mM DTT was added to quench the reaction and excess fluorophores were removed by dialyzing into buffer containing 20 mM potassium phosphate (pH 7.0) and 500 mM NaCl.

**Fluorescent labeling of live bacteria**

Post-exponential phase bacterial cultures were collected and resuspended in an M9-glucose medium supplemented with 1 μM fluorophore-conjugated protein and incubated in the dark at room temperature for 30 min. After the elimination of unbound labels by washing, the cells were resuspended in M9-glucose (100 μl). 10 μl of the cell suspension was carefully placed onto 1% (w/v) agarose pads on a microscope slide and sealed with a coverslip. The result was acquired using a high-speed rotary disc-type fluorescence confocal microscope (Andor Revolution-XD, UK) [4, 5, 12].

**Flow cytometry analysis**

Overnight cultures of *E. coli* BL21(DE3) harboring the pET28a vector or its derivatives expressing TepC, TepC-TipC or TepC^E17A^ were inoculated into fresh LB broth and induced with 0.3 mM IPTG at 26°C. Bacterial cells collected at different time points were washed, fixed and stained with the One-step TUNEL cell apoptosis detection kit (Beyotime Biotechnology, China). Genomic DNA breakage catalytically labeled with green fluorescent probe FITC were detected with flow cytometry (Beckman, CytoFLEX) and analyzed with the FlowJo_V10 software [12].

**Murine infection and competition assays *in vivo***

The protocol was approved by the Animal Welfare and Research Ethics Committee of Northwest A&F University (Protocol number: XN2023-1004). Six-week-old female mice (BALB/c) used in this study were obtained from Beijing Vital River Laboratory Animal Technology Co., Ltd. (China). The mice were housed in a controlled environment with a temperature of 24 ± 2°C, humidity of 50 ± 10%, air flow of 35 exchanges, and a light-dark cycle of 12 hours each. They were provided with ad libitum access to food and drink. Six-week-old female BALB/c mice were adapted for 3 days in the lab and orally gavaged with 10^9^ CFUs of relevant *Yptb* strains harboring the pKT100 (Km^R^) plasmid. When indicated, mice were orally gavaged with 100 μl of antibiotics cocktail (20 mg ml^-1^ Ampicillin; 10 mg ml^-1^ Vancomycin; 20 mg ml^-1^ Metronidazole; 20 mg ml^-1^ Neomycin; 0.2 mg ml^-1^ Amphotericin B) for one week prior to *Yptb* infection. Mice were sacrificed after 4 days and 6 days post-infection, and the CFUs of each *Yptb* strain in the cecum were counted on selective YLB agar plates (40 mg ml^-1^ Nalidixic acid; 50 mg ml^-1^ Kanamycin). As for *in vivo* interbacterial competition assays, mice pre-treated with the antibiotics cocktail for one week were orally gavaged with 5 × 10^8^ CFUs of recipient strains (*Escherichia coli*, *Salmonella* Typhimurium, or *Enterococcus xiangfangensis*) on day 1, and gavaged with 5 × 10^8^ CFUs of different *Yptb* donor strains on day 2. Mice were sacrificed on indicated time points, and cecum tissue was separated, serial diluted, and spread on YLB (40 mg ml^-1^ nalidixic acid; 50 mg ml^-1^ Kanamycin, for selection of *Yptb*), MacConkey (gentamicin, for selection of *E. coli*), XLT4 (for selection of *S.* Typhimurium) or *Enterococcus faecalis* agar plates (for selection of *E. xiangfangensis*) for CFU enumeration [4, 5, 12].

**Microbiota analysis**

DNA for gut microbiota analysis was extracted from the cecum content of mice treated with different strains by the PowerSoil DNA isolation kit following the manufacturer’s protocol and 16S rRNA gene high-throughput sequencing using the HiSeq PE250 platform (Illumina). Primers F341 (CCTACGGGRSGCAGCAG) and R806 (GGACTACVVGGGTATCTAATC) were used to amplify the V3-V4 regions. Amplicon sequencing bioinformatics was used with EasyAmplicon v1.0 [13]. The non-redundancy sequences are denoised into amplicon sequence variants (ASVs) with USEARCH v10.0 [14]. The RDP database was used for taxonomic annotation [15]. Diversity analysis was performed with QIIME 2 and vegan v2.5-6 package, and visualized by using the ggplot2 package in R v4.1 [16].

**Supplementary Figures**


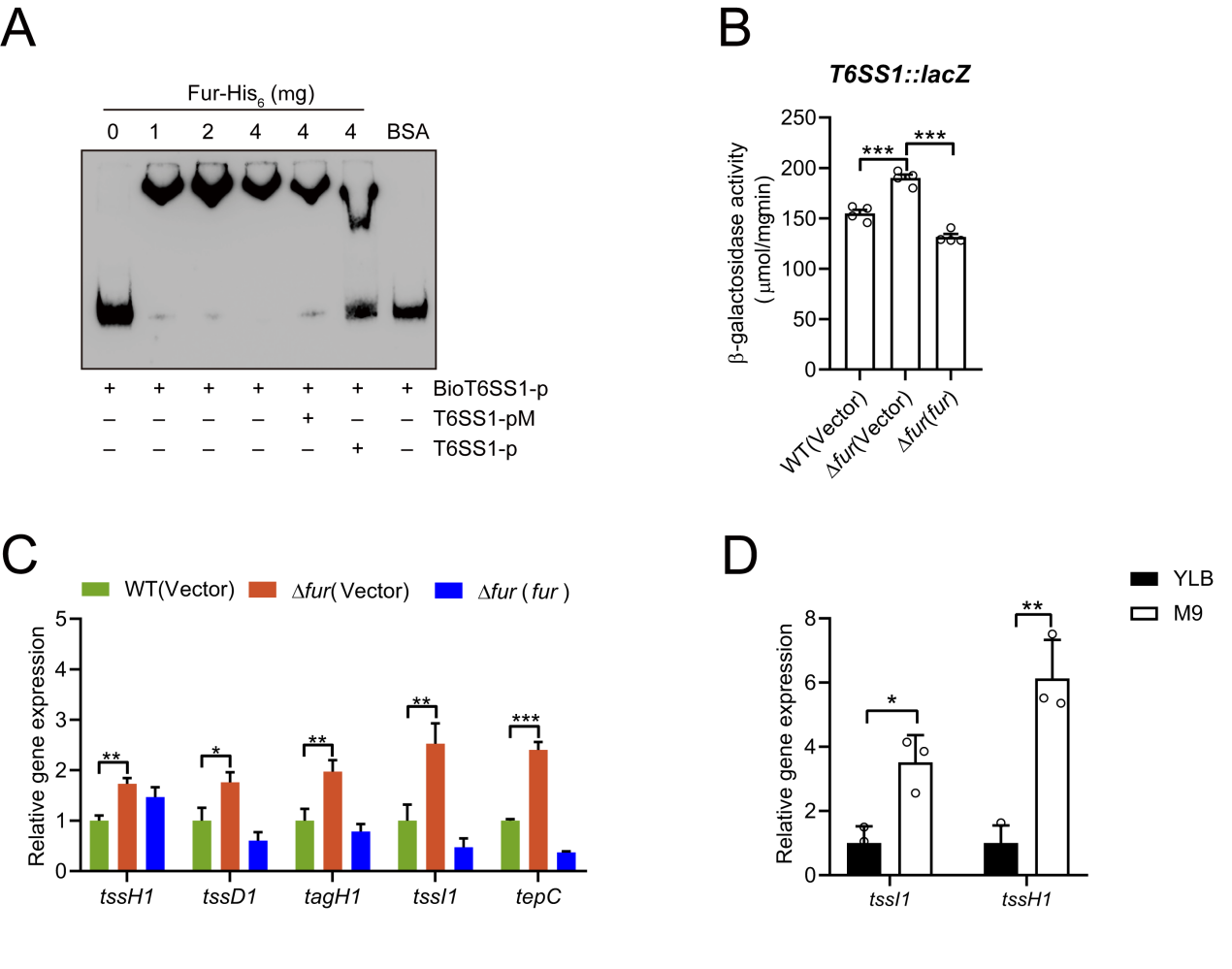


**Figure S1. The T6SS-1 is negatively regulated by Fur.**

**A**. EMSA analysis of the binding of Fur to the T6SS-1 promoter. A biotin-labeled probe containing T6SS-1 promoter region (BioT6SS1-p) was incubated with Fur to determine the binding. The protein-DNA complexes were detected by streptavidin-conjugated HRP and chemiluminescent substrates. Note that the protein-DNA complex was competitively abolished by adding an excessive unlabeled probe (T6SS1-p) but not the mutant probe (T6SS1-pM).

**B.** Fur represses the expression of T6SS-1. The β-galactosidase activities of the T6SS-1 promoter from chromosomal *lacZ* fusions in the indicated *Yptb* strains were measured.

**C.** qRT-PCR analysis of the expression levels of representative T6SS-1 genes and its downstream gene *tepC* in the *Yptb* WT, Δ*fur*, and Δ*fur*(*fur*) strains.

**D.** qRT-PCR analysis of the expression levels of representative T6SS-1 genes in YLB and M9 liquid.

Data are mean ± SD from three biological replicates. *, *p*<0.05; **, *p*<0.01; ***, *p*<0.001.


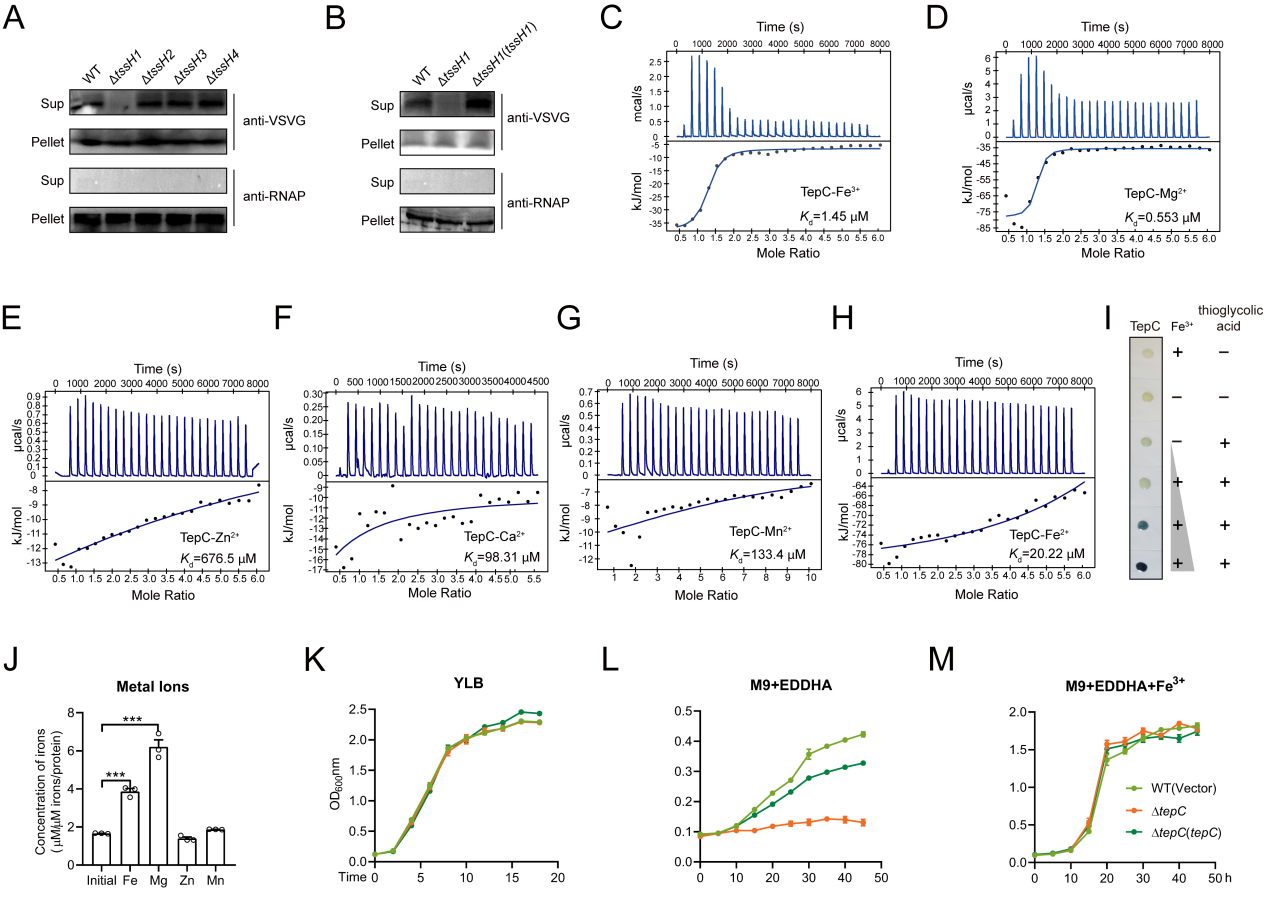


**Figure S2. The *Yptb* T6SS-1 secretes the proteinaceous siderophore TepC to mediate exploitative competition.**

**A-B.** TepC is a T6SS-1 effector. The indicated strains expressing TepC-VSVG were cultured to OD_600_ = 1.6, then total cell pellets and secreted proteins (Sup) in the culture supernatant were isolated and probed for the presence of the TepC protein with western blotting. Cytosolic RNA polymerase (RNAP) was used as a control.

**C-H.** The binding between TepC and Fe^3+^ (**C**), Mg^2+^ (**D**), Zn^2+^ (**E**), Ca^2+^ (**F**), Mn^2+^ (**G**), and Fe^2+^ (**H**) was examined with ITC. The top panel displays the heat of injection, whereas the bottom panel shows the normalized integration data as a function of the molar syringe and cell concentrations. The data were analyzed using the NanoAnalyze software (TA Instruments).

**I.** Ferene S staining assay showed the specific binding of Fe^3+^ but not Fe^2+^ by TepC. Ferene-S is a chromogenic chelator that exhibits a color change to blue when interacting with Fe^2+^ in solution. In the presence of the reducing agent thioglycolic acid, positive Ferene S staining for iron was observed for iron-containing TepC but not for the iron-free TepC treated by EDDHA. In the absence of the reducing agent thioglycolic acid, iron-containing TepC did not stain positive with Ferene S, proving that the bound iron is Fe^3+^ rather than Fe^2+^.

**J.** The detection of divalent ion binding by TepC was accomplished with atomic absorption spectrometry.

**K-M.** The growth of indicated *Yptb* strains in YLB (**K**), M9 containing 40 μM EDDHA (**L**) or M9 containing 40 μM EDDHA and 40 μM Fe^3+^ (**M**), respectively. Cell growth was monitored by measuring OD_600_.


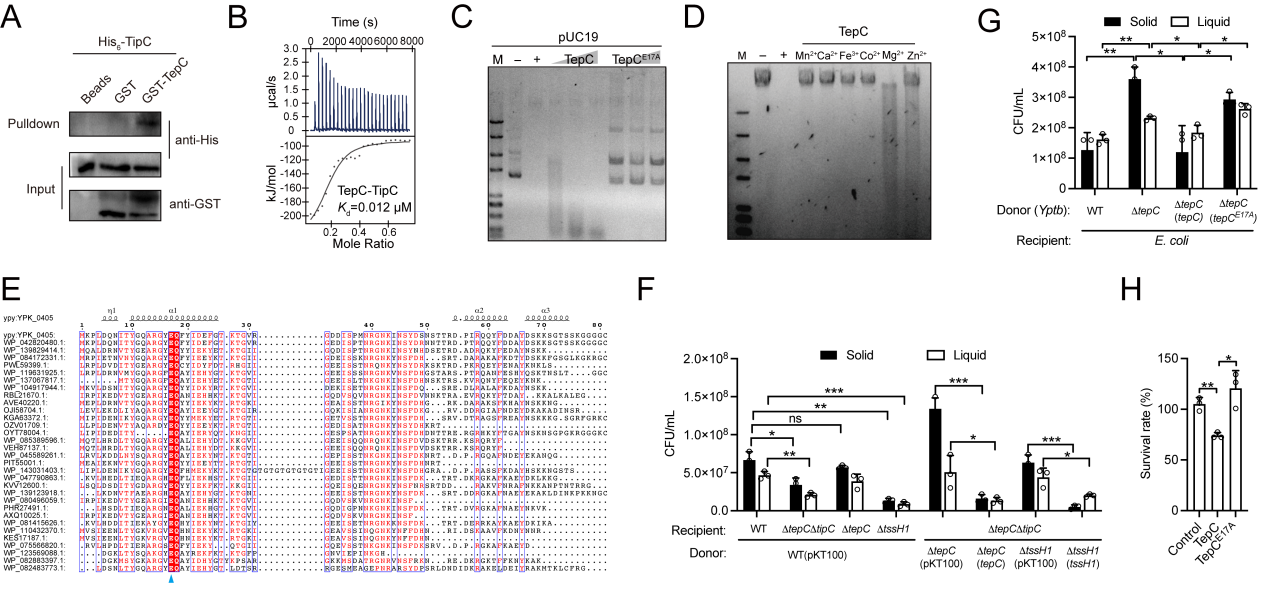


**Figure S3. TepC mediates interference competition via Mg^2+^-dependent DNase activity.**

**A.** Direct binding between TepC and TipC was determined with a GST pull-down assay. His_6_-TipC was incubated with beads only, GST, or GST-TepC. The protein complexes captured on glutathione beads were detected by western blotting.

**B.** The interaction between TepC and TipC was determined by ITC. The data were analyzed using the NanoAnalyze software (TA Instruments).

**C.** DNase assays indicating the integrity of pUC19 co-incubated without (-) or with the DNase I control (+), TepC (0.25, 0.5, 1 μM), or TepC^E17A^ (0.25, 0.5, 1 μM) at 37°C for 30 min. Reaction products were analyzed using agarose gel electrophoresis.

**D.** Effects of metal ions on the DNase activity of TepC. λ DNA was incubated without (-) or with the DNase I control (+) or TepC in the presence of Mn^2+^, Ca^2+^, Fe^3+^, Co^2+^, or Mg^2+^ (2 mM) in the DNase reaction buffer for 30 min at 37°C. The integrity of DNA was analyzed on a 0.7% agarose gel.

**E.** Alignment of TepC with orthologs. The ESPript output was acquired from the NCBI BlastP database and aligned using the CLUSTAL-2.1 algorithm. Conserved residues are indicated in red. The highly conserved E17 residue was pointed out with a blue triangle.

**F.** Intraspecies competition between the indicated *Yptb* donor and recipient strains in M9 medium. An equal amount of donor and recipient strains were mixed and grown on a solid or liquid medium for 24 h at 30°C. The CFU of recipient strains was measured based on plate counts.

**G.** Interspecies competition between the donor strain *Yptb* and recipient strain *E. coli*. An equal amount of donor and recipient strains were mixed and grown on a solid or liquid medium for 24 h at 30°C. The CFU of recipient strains was measured based on plate counts.

**H.** Toxicity assays of purified TepC protein. Stationary-phase cultures of *E. coli* were diluted 40-fold in M9 liquid and treated with purified TepC or TepC^E17A^ (0.1 mg ml^−1^) for 1 h. The survival rates of bacterial cells were determined by counting the CFUs after treatment.

Data in **F**-**H** are mean ± SD from three biological replicates. *, *p*<0.05; **, *p*<0.01; ***, *p*<0.001.

**
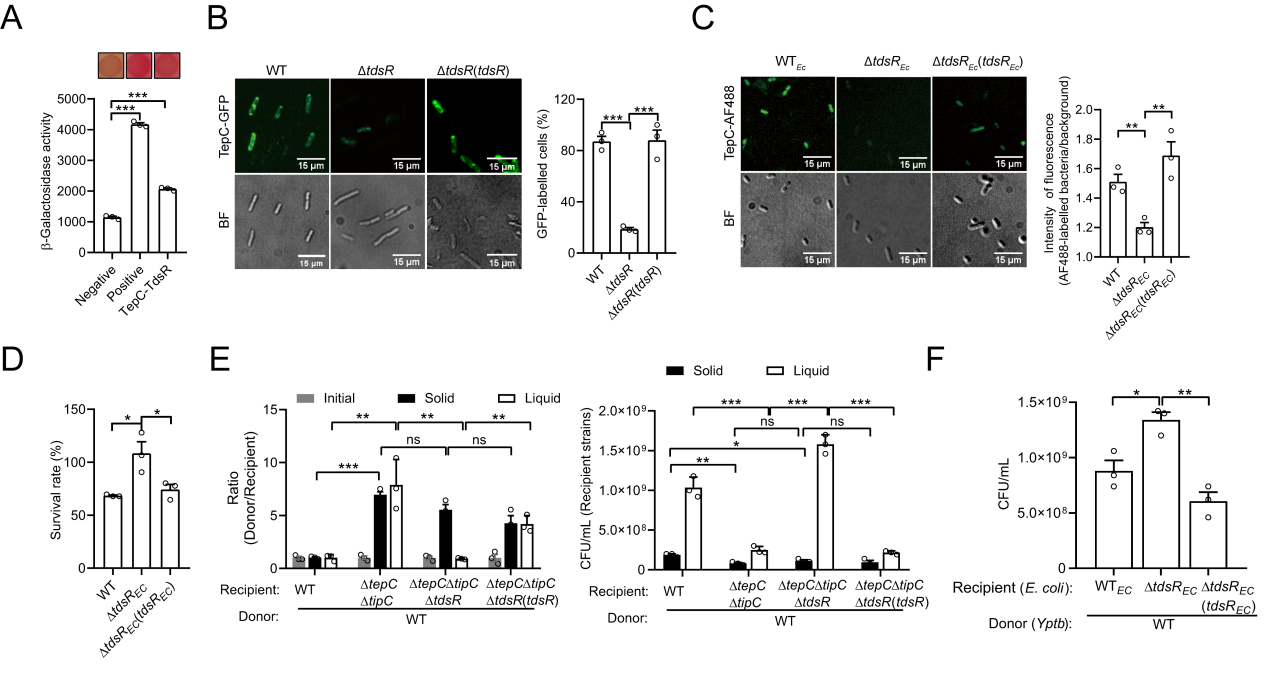
**

**Figure S4. TdsR is the outer membrane receptor of TepC and mediates the interference competition.**

**A**. Verify the interaction between TepC and TdsR with the bacterial two-hybrid assay. Interactions were visualized with the MacConkey maltose plates (upper) and quantified with the β-galactosidase assay (lower).

**B.** Fluorescence labeling of relevant *Yptb* strains with TepC-GFP (left) and their corresponding quantifications of binding (right). Scale bars: 15 μm.

**C.** Effects of *E. coli* TdsR*_Ec_* on the cell entry of TepC. Fluorescence labeling of *E. coli* WT*_Ec_*, Δ*tdsR_Ec_*, and Δ*tdsR_Ec_(tdsR_Ec_)* strains with TepC-AF488 (left) and its quantification (right). Scale bars: 15 μm.

**D.** Effects of TdsR*_Ec_* on the toxicity of TepC to *E. coli*. Stationary-phase cultures of *E. coli* WT*_Ec_*, Δ*tdsR_Ec_*, and Δ*tdsR_Ec_(tdsR_Ec_)* strains were diluted 40-fold in M9 liquid, then treated with purified TepC (0.1 mg ml^−1^) for 1 h. The survival rate of bacteria was determined by counting the CFUs after treatment.

**E**. Intraspecies competition assays between the *Yptb* WT donor and indicated recipient strains. Donor and recipient strains were mixed 1:1 and then grown for 24 h on a M9 solid or liquid medium at 30°C. Left: the competitive index of the donor and recipient strains was calculated based on plate counts. Right: the CFU of recipient strains was measured based on plate counts.

**F.** Interspecies competition assays between the *Yptb* WT donor and *E. coli* WT*_Ec_*, Δ*tdsR_Ec_*, or Δ*tdsR_Ec_(tdsR_Ec_)* recipient, respectively. Donor and recipient strains were mixed 1:1 and then grown for 24 h in M9 liquid at 30°C. The CFU of recipient strains was measured based on plate counts.

Data are mean ± SD from three biological replicates. *, *p*<0.05; **, *p*<0.01; ***, *p*<0.001.


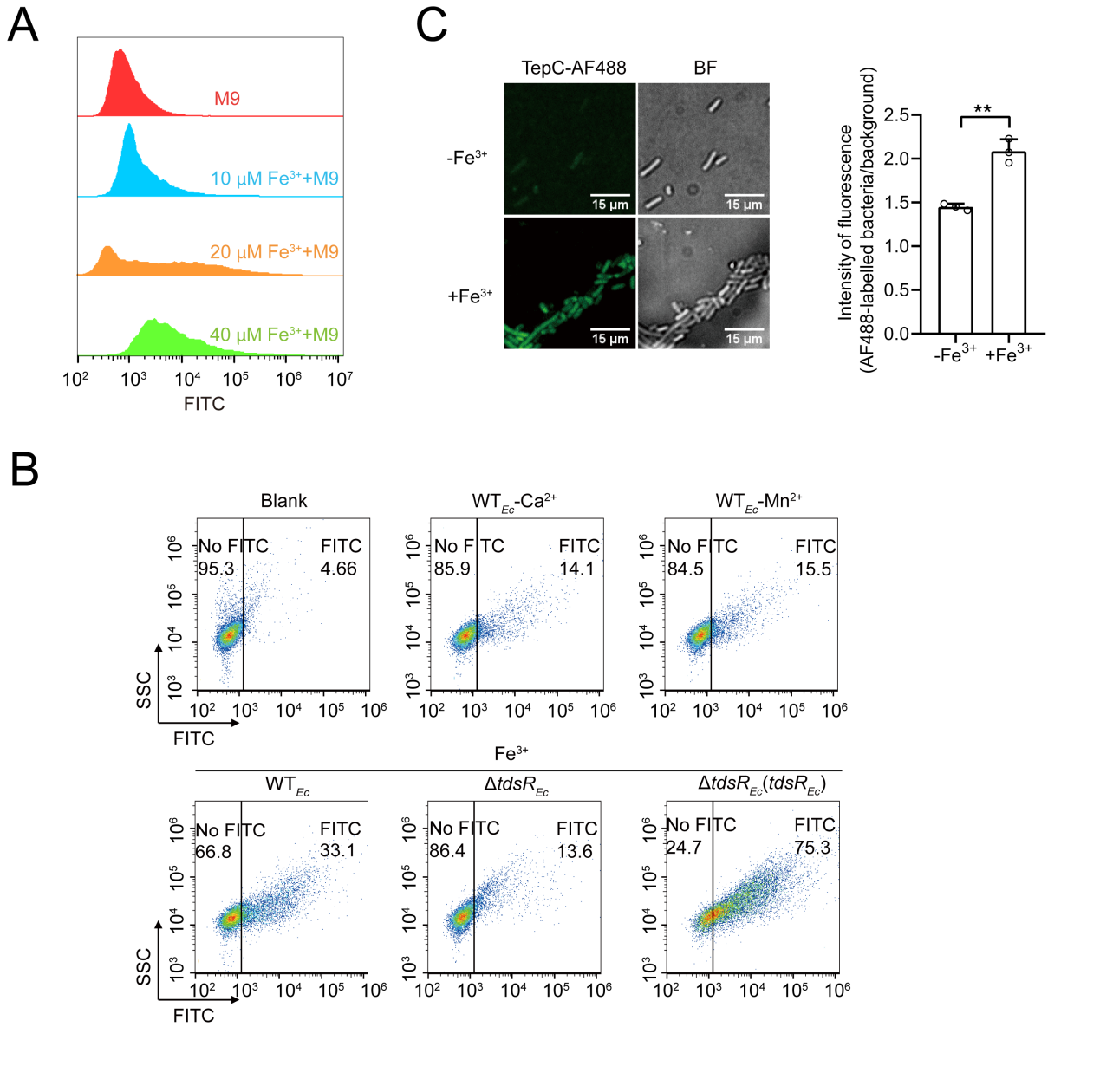


**Figure S5. Fe^3+^ promotes the binding and internalization of TepC.**

**A**. Flow cytometry monitors the binding of TepC-GFP to *E. coli* cells in the M9 medium containing increasing concentrations of Fe^3+^ (0-40 μM).

**B.** The binding between TepC-GFP and *E. coli* cells depends on the presence of the TdsR*_Ec_* receptor. TepC-GFP proteins were incubated with *E. coli* WT*_Ec_*, Δ*tdsR_Ec_*, or Δ*tdsR_Ec_*(*tdsR_Ec_*) cells, respectively, in the presence of Fe^3+^, Ca^2+^, or Mn^2+^ (20 μM) in M9. The Blank is the WT strain in the M9 medium without adding metal ions. After 30 min incubation, samples were washed with M9 three times to remove unbound TepC-GFP. The capability of binding was detected by flow cytometry.

**C.** Fluorescence labeling of *E. coli* cells with TepC-AF488 in the absence or presence of Fe^3+^ (20 μM, left) and its corresponding quantification (right). Scale bars: 15 μm.

Data are mean ± SD from three biological replicates. **, *p*<0.01.


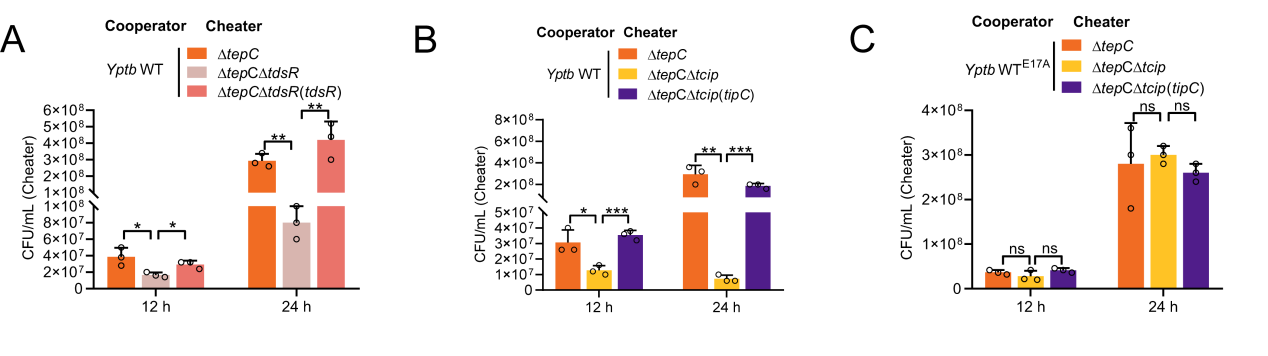


**Figure S6. Roles of TdsR, TipC and the DNase activity of TepC in cheating and policing.**

**A-C.** *In vitro* cheating and policing experiments between indicated *Yptb* cooperator and cheater strains were performed by mixing corresponding cooperator and cheater strains equally in M9 medium and incubated at 30°C. The CFUs of each cheater strain were counted on selective plates containing appropriate antibiotics at indicated time points.

**A.** Exploitation of TepC requires the TdsR receptor. *Yptb* WT was used as a cooperator, and Δ*tepC*, Δ*tepC*Δ*tdsR*, and Δ*tepC*Δ*tdsR*(*tdsR*) were used as cheaters.

**B.** The immunity protein TipC is required to exploit TepC. *Yptb* WT was used as a cooperator, and Δ*tepC*, Δ*tepC*Δ*tipC*, and Δ*tepC*Δ*tipC*(*tipC*) were used as cheaters.

**C.** The DNase activity of TepC is required for policing cheaters. *Yptb* WT^E17A^ was used as a cooperator, and Δ*tepC*, Δ*tepC*Δ*tipC*, and Δ*tepC*Δ*tipC*(*tipC*) were used as cheaters.

Data are mean ± SD from three biological replicates. *, *p*<0.05; **, *p*<0.01; ***, *p*<0.001; ns, no significance.


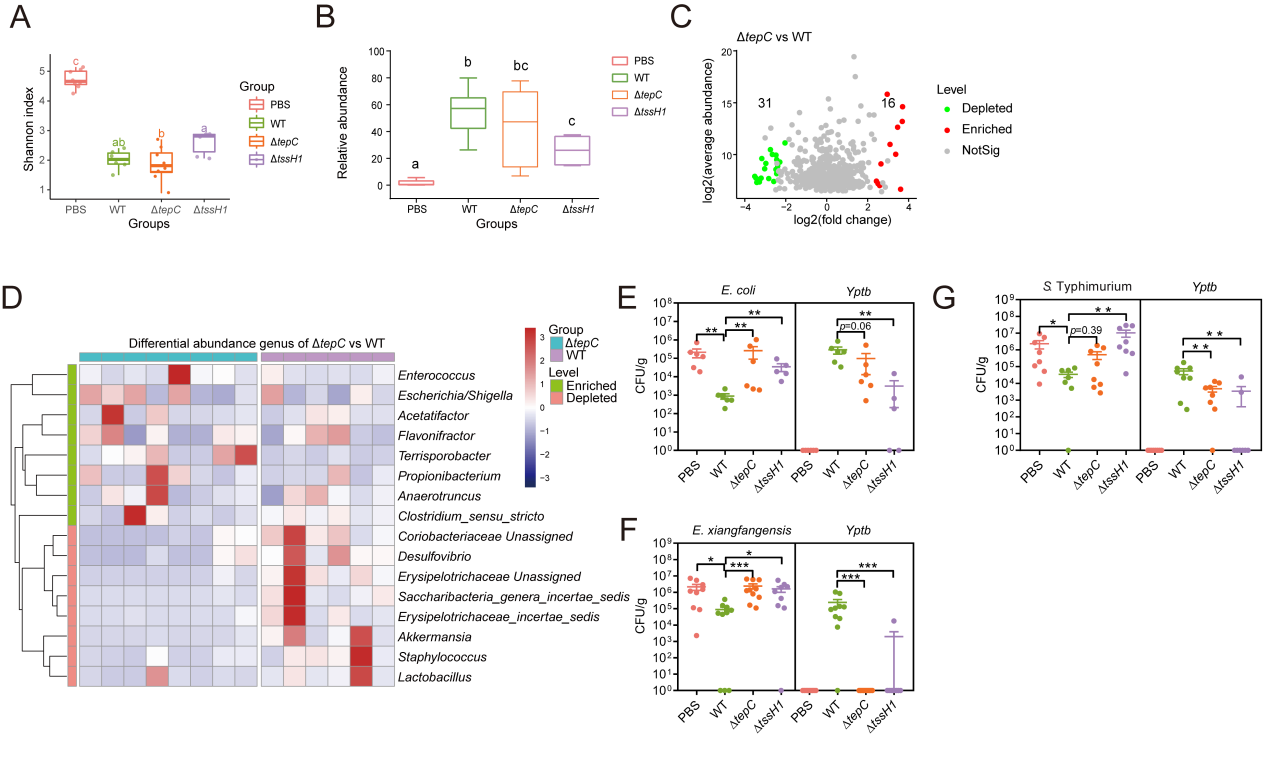


**Figure S7. The analysis of 16S rRNA gene amplicon of cecal contents of experimental mice (n > 5 for each group) and the competition assays in *vivo*.**

**A**. Alpha diversity of the gut microbiota with the Shannon index in the four treatments. The horizontal bars within boxes represent medians. Different letters above the whiskers denote significant differences between each group determined with analysis of variance tests (*p* < 0.05). Different groups that have the same letter have no statistical difference.

**B.** Relative abundance (RA) of *Yptb* in the four treatments (Wilcoxon test, *p* < 0.05).

**C.** Volcano plot showing ASVs enriched and depleted in the Δ*tepC* group compared with the WT group (*p* < 0.05, abundance > 0.01%). Each point represents an individual ASV and the position along the X-axis represents the abundance fold change.

**D.** Differential gut microbial genus between the WT group and the Δ*tepC* group (*p* < 0.05, abundance > 0.01%).

**E-G.** *In vivo* competition assays between indicated *Yptb* donor strains and recipient strains. Antibiotics-pretreated mice (n = 5-10) were inoculated with *E. coli* (**E**), *S.* Typhimurium (**F**), and *E. xiangfangensis* (**G**) on day 1, and an equal amount of the indicated *Yptb* strains were orally gavaged after 24 h (with PBS as a buffer control). Mice were sacrificed on day 4, and CFUs of each bacterium in the cecum were counted on selective plates. Statistical analysis of experiments in **E**-**G** was carried out using the Mann–Whitney test. *, *p*<0.05; **, *p*<0.01; ***, *p*<0.001.


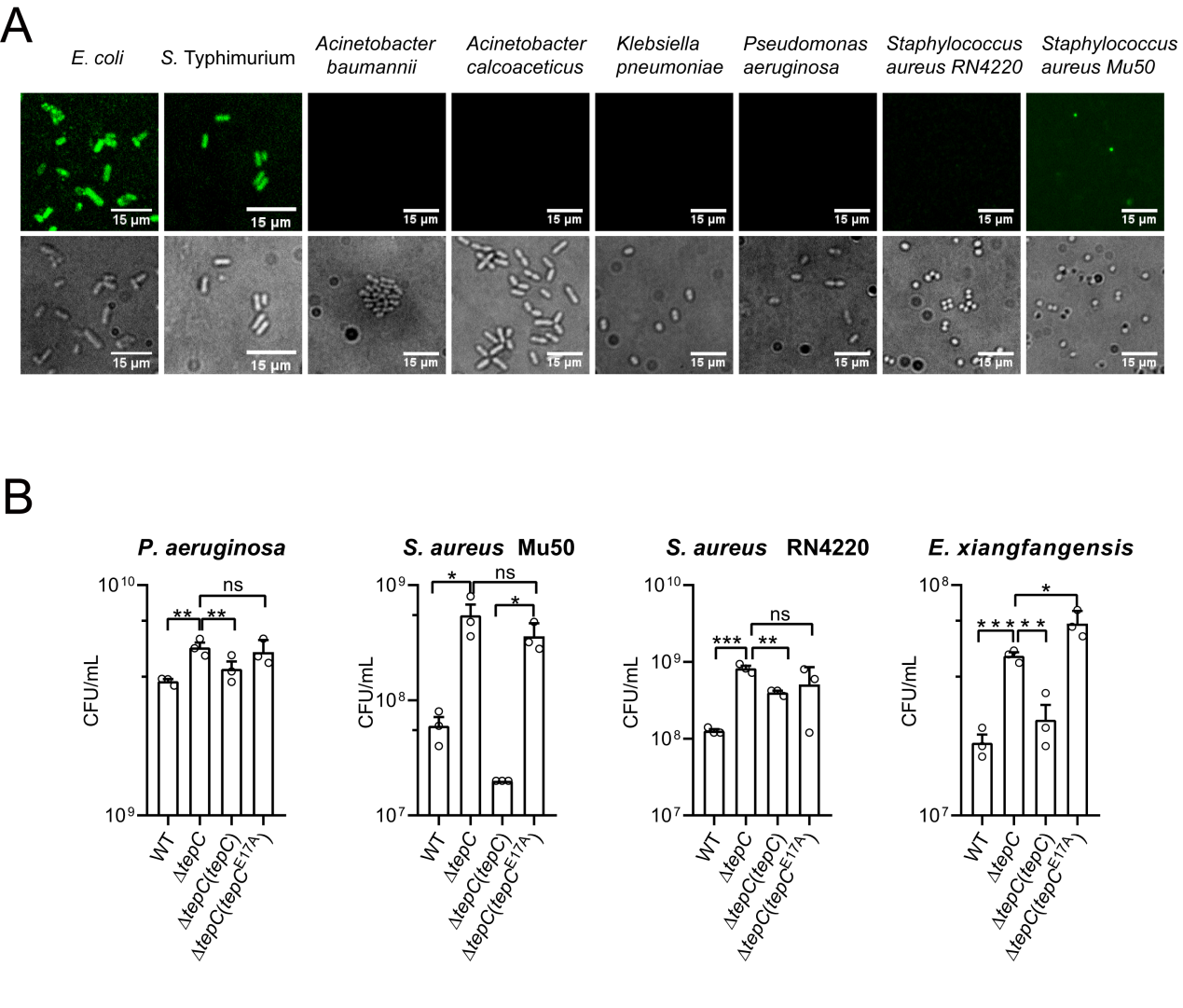


**Figure S8. The ability of TepC to mediate contact-dependent interference competition.**

**A.** Fluorescence labeling of indicated bacterial strains with TepC-AF488 to assess the cell entry ability of TepC to different bacterial cells. Scale bars: 15 μm.

**B.** Contact-dependent interspecies competition assays between the indicated *Yptb* donors and *P. aeruginosa*, *S. aureus* Mu50, *S. aureus* RN4220, and *E. xiangfangensis* recipients. Equal CFUs of donor and recipient strains were mixed evenly and grown for 24 h in the M9 medium. The survival of recipient cells was quantified by counting CFUs on corresponding selective plates.

Data are mean ± SD from three biological replicates. *, *p*<0.05; **, *p*<0.01; ***, *p*<0.001.

**Supplementary References**

1. Zhu, L., L. Xu, C. Wang, C. Li, M. Li, Q. Liu, et al., T6SS translocates a micropeptide to suppress STING-mediated innate immunity by sequestering manganese*.* *Proc Natl Acad Sci U S A*, 2021. **118**(42): e2103526118.

2. Zhang, L., M. Li, Q. Li, C. Chen, M. Qu, M. Li, et al., The Catabolite Repressor/Activator Cra Is a Bridge Connecting Carbon Metabolism and Host Colonization in the Plant Drought Resistance-Promoting Bacterium Pantoea alhagi LTYR-11Z*.* *Appl Environ Microbiol*, 2018. **84**(13).

3. Jiang, Y., B. Chen, C. Duan, B. Sun, J. Yang, and S. Yang, Multigene editing in the Escherichia coli genome via the CRISPR-Cas9 system*.* *Appl Environ Microbiol*, 2015. **81**(7): 2506-14.

4. Song, L., J. Pan, Y. Yang, Z. Zhang, R. Cui, S. Jia, et al., Contact-independent killing mediated by a T6SS effector with intrinsic cell-entry properties*.* *Nat Commun*, 2021. **12**(1): 423.

5. Si, M., C. Zhao, B. Burkinshaw, B. Zhang, D. Wei, Y. Wang, et al., Manganese scavenging and oxidative stress response mediated by type VI secretion system in *Burkholderia thailandensis.* *Proc Natl Acad Sci U S A*, 2017. **114**(11): E2233-E2242.

6. Lin, J., W. Zhang, J. Cheng, X. Yang, K. Zhu, Y. Wang, et al., A *Pseudomonas* T6SS effector recruits PQS-containing outer membrane vesicles for iron acquisition*.* *Nat Commun*, 2017. **8**: 14888.

7. Wang, T., M. Si, Y. Song, W. Zhu, F. Gao, Y. Wang, et al., Type VI Secretion System Transports Zn^2+^ to Combat Multiple Stresses and Host Immunity*.* *PLoS Pathog*, 2015. **11**(7): e1005020.

8. Shen, X., S. Banga, Y. Liu, L. Xu, P. Gao, I. Shamovsky, et al., Targeting eEF1A by a *Legionella pneumophila* effector leads to inhibition of protein synthesis and induction of host stress response*.* *Cell Microbiol*, 2009. **11**(6): 911-26.

9. Karimova, G., J. Pidoux, A. Ullmann, and D. Ladant, A bacterial two-hybrid system based on a reconstituted signal transduction pathway*.* *Proc Natl Acad Sci U S A*, 1998. **95**(10): 5752-6.

10. Li, C., L. Zhu, D. Wang, Z. Wei, X. Hao, Z. Wang, et al., T6SS secretes an LPS-binding effector to recruit OMVs for exploitative competition and horizontal gene transfer*.* *ISME J*, 2022. **16**(2): 500-510.

11. Doyle, C.K., X. Zhang, V.L. Popov, and J.W. McBride, An immunoreactive 38-kilodalton protein of *Ehrlichia canis* shares structural homology and iron-binding capacity with the ferric ion-binding protein family*.* *Infect Immun*, 2005. **73**(1): 62-9.

12. White, P., A. Joshi, P. Rassam, N.G. Housden, R. Kaminska, J.D. Goult, et al., Exploitation of an iron transporter for bacterial protein antibiotic import*.* *Proc Natl Acad Sci U S A*, 2017. **114**(45): 12051-12056.

13. Liu, Y.X., Y. Qin, T. Chen, M. Lu, X. Qian, X. Guo, et al., A practical guide to amplicon and metagenomic analysis of microbiome data*.* *Protein Cell*, 2021. **12**(5): 315-330.

14. Edgar, R.C., Search and clustering orders of magnitude faster than BLAST*.* *Bioinformatics*, 2010. **26**(19): 2460-1.

15. Cole, J.R., Q. Wang, J.A. Fish, B. Chai, D.M. McGarrell, Y. Sun, et al., Ribosomal Database Project: data and tools for high throughput rRNA analysis*.* *Nucleic Acids Res*, 2014. **42**(Database issue): D633-42.

16. Bolyen, E., J.R. Rideout, M.R. Dillon, N.A. Bokulich, C.C. Abnet, G.A. Al-Ghalith, et al., Reproducible, interactive, scalable and extensible microbiome data science using QIIME 2*.* *Nat Biotechnol*, 2019. **37**(8): 852-857.
